# Marine Natural Products from New Caledonia—A Review

**DOI:** 10.3390/md14030058

**Published:** 2016-03-16

**Authors:** Sofia-Eléna Motuhi, Mohamed Mehiri, Claude Elisabeth Payri, Stéphane La Barre, Stéphane Bach

**Affiliations:** 1 sofia-elena.motuhi@ird.frclaude.payri@ird.fr; 2; 3 mohamed.mehiri@unice.fr; 4

**Keywords:** New Caledonia, marine biodiversity, bioactive molecules pipeline, chemodiversity, coral reefs

## Abstract

Marine micro- and macroorganisms are well known to produce metabolites with high biotechnological potential. Nearly 40 years of systematic prospecting all around the New Caledonia archipelago and several successive research programs have uncovered new chemical leads from benthic and planktonic organisms. After species identification, biological and/or pharmaceutical analyses are performed on marine organisms to assess their bioactivities. A total of 3582 genera, 1107 families and 9372 species have been surveyed and more than 350 novel molecular structures have been identified. Along with their bioactivities that hold promise for therapeutic applications, most of these molecules are also potentially useful for cosmetics and food biotechnology. This review highlights the tremendous marine diversity in New Caledonia, and offers an outline of the vast possibilities for natural products, especially in the interest of pursuing collaborative fundamental research programs and developing local biotechnology programs.

## 1. Introduction

Located approximately at 165° E and 21°30′ S, the New Caledonia archipelago enjoys a privileged position east of the city of Rockhampton, central province of the Great Barrier Reef, 1500 km away from the Australian Queensland coast, with surface seawater temperatures mild enough to avoid recurrent coral bleaching episodes.

**Figure 1:**
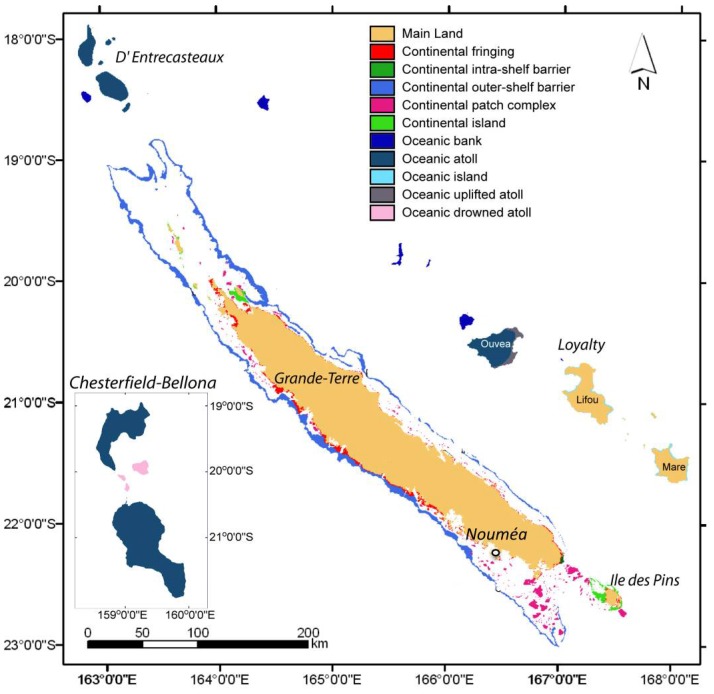
The archipelago of New Caledonia and its reef complexes (adapted from Andréfouët *et al.* 2009 [2], with kind permission from the author).

This tropical subregion of Melanesia is a relic of a continental landmass that drifted away from the super-continent Gondwana at the end of the Cretaceous period (see Pelletier 2007) [1]. Successive geological events have resulted in an archipelago that comprises a main island called Grande Terre, Ile des Pins in the south, the Entrecasteaux reefs in the north, the Loyalty Islands in the east and, finally, the Chesterfield-Bellona plateau in the west, located at mid-distance to Australian coast (Figure 1).

New Caledonian waters represent an exclusive economic zone (EEZ) spanning more than 1,700,000 km^2^. The EEZ includes a vast and complex reef system of 4537 km^2^ of reef area and 31,336 km^2^ of non-reef area [2] and represents the second most important coral reef complex and the longest continuous barrier reef in the world, stretching over 1600 km.

The reef systems together make up a unique marine ecosystem [3] with more than 161 different reef units [4] supporting high marine species diversity and abundance [5], including so-called “living fossils” such as a stalked crinoid and a shelled cephalopod.

Only since the 19th century has attention been paid to diversity of the New Caledonian reef systems. Many expeditions have been conducted to study the oceanic life of these hitherto unexplored oceanic zones. The first oceanographic cruise, the *Challenger* expedition (1872–1876), led to the collection of thousands of previously unknown marine species [6]. Since the 19th century, scientific interest has shifted from the study of “exotic” specimens stored in museum collections and classified according to their morphological features, to the study and understanding of marine ecosystems. Charles Darwin’s landmark expeditions in the Eastern Pacific islands and his remarkable observations on the formation of atolls from subsiding volcanic islands sparked continued interest in ecosystems biology in oceans across the world. Research vessels are now equipped with sophisticated equipment to carry out on-site sample analysis (e.g., molecular biology) and data transmission via satellite systems. For example, the schooner *Tara*, specially fit out with on-board facilities, recently sailed around the world to study the impact of global warming on plankton and coral reef systems across the Pacific [7].

Pioneering scientific investigations in New Caledonian waters were carried out locally after World War II. The naturalist René Catala initiated ecological surveys and species census around Ile aux Canards [8], an island off Nouméa now directly exposed to human activities, with recent changes in reef zonation and species composition. In addition, in 1956, Catala founded the Aquarium of Nouméa (now the *Aquarium des Lagons*), a first-of-its-kind structure specifically designed to observe rare and fragile marine species in their environment, including homegrown corals. Catala incidentally discovered the fluorescence of living corals [9] and other invertebrates (fluorescence appears to be a photoprotective and possibly temperature-regulating mechanism). A few years later, the Singer-Polignac expedition (1960–1963) explored St. Vincent Bay and the east coast of Grande Terre [10,11,12]. Similarly, the American ecologist, Arthur Lyon Dahl, a regional adviser at the South Pacific Commission from 1974 to 1982, demonstrated the importance of surface area in ecological analysis and described several methods for conducting surveys of coral reefs [13,14].

**Figure 2:**
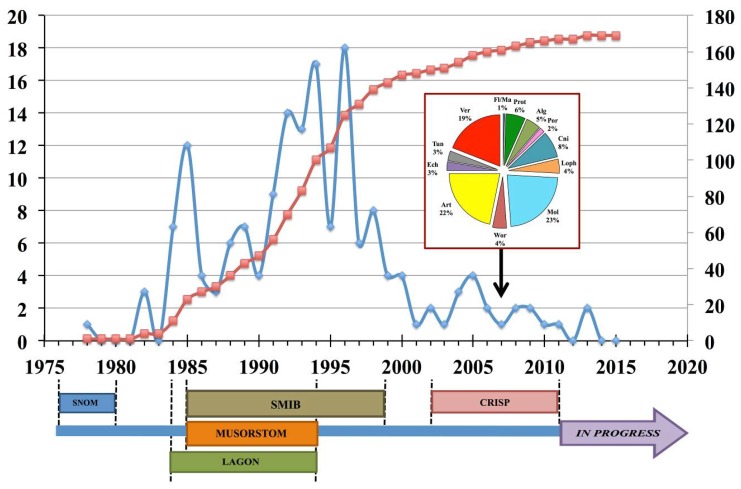
Publications in scientific journals on organisms collected within various research programs. Cumulative data representing the total number of publications generated since 1976 to present (red line) and the number of publications produced per year since 1976 (blue line). The timeline on the x-axis shows the various regional marine biodiversity programs spanning the 40-year period. These statistics do not include reports, reviews and book chapters. The insert shows the relative percentages of major taxon investigated up to 2007 [5]. **SNOM**: *Substances Naturelles d’Origine Marine*; **SMIB**: *Substances Marines d’Intérêt* *Biologique*; **LAGON**; **MUSORSTOM** (now Tropical Deep-Sea Benthos): acronym for the joint expeditions of the National Museum of Natural History (MNHN) and the *Office de la* *Recherche Scientifique et Technique d’Outre-Mer* (ORSTOM, now IRD); **CRISP**: Coral Reef InitiativeS for the Pacific; ***in progress****:* various research programs including chemical and pharmacological investigations on micro- and macroalgae. **Fl/Ma**: Flora and Marine Angiosperms; **Pro**: Protozoa; **Alg**: Algae; **Por**: Porifera; **Cni**: Cnidaria; **Loph**: Lophophorates; **Mol**: Mollusks; **Wor**: Worms; **Art**: Arthropoda; **Ech**: Echinodermata; **Tun**: Tunicata; **Ver**: Vertebrata.

Bioprospecting studies in New Caledonia were initiated in 1976 by Pierre Potier from the ICSN (Institute of Natural Substances Chemistry, Gif-sur-Yvette, mainland France) as part of the national research program SNOM (*Substances Naturelles d’Origine Marine*) and benefited from collaboration with scientific divers from IRD (*Institut de Recherche pour le Développement*, ex. ORSTOM, *Office de la Recherche Scientifique et Technique d’Outre-Mer*) in Nouméa (Figure 2). SNOM set out to explore marine biodiversity extensively with the taxonomic expertise from the National Museum of Natural History (MNHN) of Paris, to identify novel molecules and to assess their biological activities primarily for pharmaceutical purposes. Collecting efforts focused mainly on invertebrates, including octocorals, porifers, echinoderms, mollusks and ascidians. Little attention has been given to macroalgae due to the lack of taxonomic support.

In 1985, the SNOM program was followed by the SMIB program (*Substances Marines d’Intérêt Biologique*), which was conducted jointly by ORSTOM and the *Centre National de la Recherche Scientifique* (CNRS), with a large collaborative network of public research institutions, e.g., the *Centre d’Etudes Nucléaires* *de Saclay*, the *Institut National de la Santé et de la Recherche Médicale* (INSERM), the MNHN, French and foreign universities, as well as several private companies (in particular, Pierre Fabre and Rhône-Poulenc).

The raw compounds and their fractions were extracted at the ORSTOM center in Nouméa and were originally sent for purification and structural determination to the ICSN (mainland France), and the biological screening was assigned to various mainland French laboratories. Gradually, separation and purification, as well as preliminary testing and benchtop assays, were carried out locally in New Caledonia at the ORSTOM (and later IRD) center, to avoid unnecessary duplication and to better target specific requests from collaborating partners. During this time, a number of candidate molecules were identified for their anticancer, cardiovascular or neurological interest (reviewed in [15]), prompting an extension of the research program and redefinition of the terms of scientific collaboration, now open to international experts. The purchase of a larger research vessel, the R/V *Alis*, in 1992, provided the opportunity to extend the bioprospecting range by dredging to 600 m deep and to access new biological resources.

The SNOM and SMIB programs led to an extensive survey of the marine biodiversity of the New Caledonian archipelago, providing many *in situ* observations and records of new taxon, which have been published in several papers [5] and illustrated in field guides, especially on sponges [16], echinoderms [17], gorgonians [18], ascidians [19], marine snakes [20], and fishes [21]. Among the 300 organisms studied, only 50 have been the object of chemical and therapeutic research and several original molecules have been described and tested on tumor development [22].

After the SNOM and SMIB programs, pharmacological bioprospecting in New Caledonia was integrated in the LAGON and MUSORSTOM biodiversity projects until 2000. Thereafter, research activities were extended to other countries in the Pacific region as part of the Coral Reef InitiativeS for the Pacific (CRISP), with emphasis on legal agreements and economic benefits to host countries.

In the meantime, some research activity focused on ciguatera and cyanobacteria toxicity after several severe poisoning events occurred in New Caledonia. In addition to pharmaceutical activities, natural marine compounds inspired new scientific approaches including chemotaxonomy and chemical ecology of benthic macroalgae to understand how opportunistic algae colonize living coral. These activities are conducted by the CoReUs/ENTROPIE research team at IRD (Figure 2). These projects are mentioned in Moretti *et al.*, (1993) [22] and detailed in a comprehensive review of novel chemical structures and associated pharmacological activities described by Laurent and Pietra published in 2004 [15]. Another review emphasizes the developmental aspects of marine molecules from South Pacific zone including New Caledonia [23].

Here, we provide an updated review of 40 years of exploration of the marine micro-/macrophyte and invertebrate chemodiversity of this species-rich zone of the Southwest Pacific, with its pharmacological potential and its ecological significance. After a description of the basic operational aspects of discovering marine natural products, an overview of the work on each major taxon is presented, illustrated by case studies that have been the highlights of the abovementioned programs for the last 40 years, and carried out locally by experts in full compliance with existing regulations of biodiversity protection and the sustainability of valuable natural resources.

## 2. Taxonomy

SCUBA diving allows visual exploration of shallow-water marine biota, making it possible not only to collect material at depths down to 60 m, but also to take photographs and record ecological information, e.g., interactions between organisms that can be useful for selecting organisms to collect and subsequent biological tests. Upon the development of blind transect dredging with limited biomass sampling on soft bottoms, collection efforts were extended to deeper zones (down to 600 m), thus sampling entirely different organisms.

### 2.1. Sample Collection Sites

Collection sites were selected to cover the large diversity of habitats ranging from shallow lagoons to deep parts of the outer slopes of the barrier reefs. They include hard and sandy bottoms, sheltered and exposed areas of the mainland (Grande Terre), Loyalty Islands, as well as remote reefs and atolls (Entrecasteaux, Chesterfield) and seamounts (Figure 1).

Each site was georeferenced and described using geomorphological and biological descriptors. Historical data have been sorted, standardized and stored along with recent information in the dedicated database LagPlon [24].

### 2.2. Biological Material Sampling

For all the groups collected for chemical purposes, specimens from each taxon were sampled for taxonomical identification and preserved in ethanol as vouchers after labelling. Collections were duplicated, one sample was kept for the IRD reference collection to facilitate new sampling efforts and the duplicate was sent to specialists for taxonomy work. *In situ* macrophotographs were taken as well as additional *ex situ* laboratory photographs when necessary. Specimens used to describe new species (holotypes) have been deposited in various museum collections in Australia (Northern Territory Museum, Darwin and Queensland Museum of South Brisbane), New Zealand, mainland France (National Museum of Natural History, Paris), Belgium (Université Libre de Bruxelles). Paratypes were systematically filed in the IRD reference collection in Nouméa, along with photographs and field records.

## 3. Chemistry

### 3.1. Biological Material

Chemical characterization and biological assays were initially carried on samples with wet weights ranging from 300 to 3000 g. However, recent progress in analytical techniques allows molecular inventory and pharmacological tests from smaller amounts of material in compliance with international regulations on the use of biological material for research purposes.

**Figure 3:**
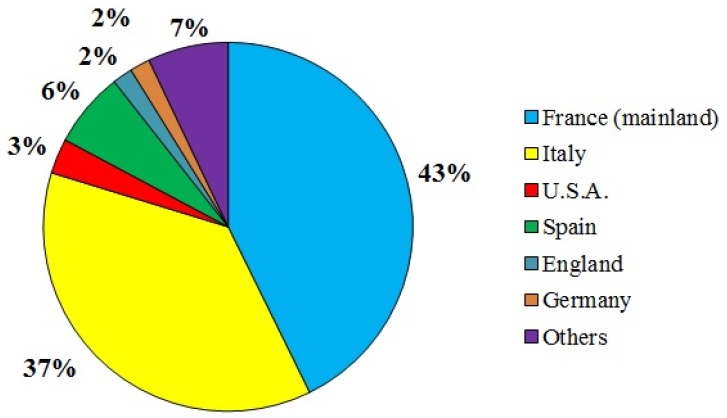
Collaborative network of chemists and pharmacologists from 1976 to 2013 as compiled individually from journal publications.

Below, we describe the basic protocols used locally at the IRD laboratories in Nouméa. Each of the molecules that have been investigated locally or with national and international partners have been treated separately (Figure 3). It is beyond the scope of this review to detail each protocol individually; they can be found in the original research papers, or in a natural products database.

### 3.2. Conditioning Samples for Chemistry

Freshly collected material is sorted by taxon and frozen on board at −20 °C, or at least placed in 70% ethanol in distilled water (slightly acidified to avoid oxidation of polar compounds) until it can be deep frozen and freeze-dried for subsequent use. Material used for enzymology or genome studies is snap-frozen on board (liquid nitrogen or dry ice in pure alcohol). For short collecting sessions (less than one full day), the material may be kept alive if each item is appropriately handled.

Back at the laboratory, the material must be ground/chopped/crushed then freeze-dried or ethanol-preserved and stowed away for later use, or sent abroad to partner laboratories.

### 3.3. Extraction

#### 3.3.1. Routine Procedure

**Figure 4:**
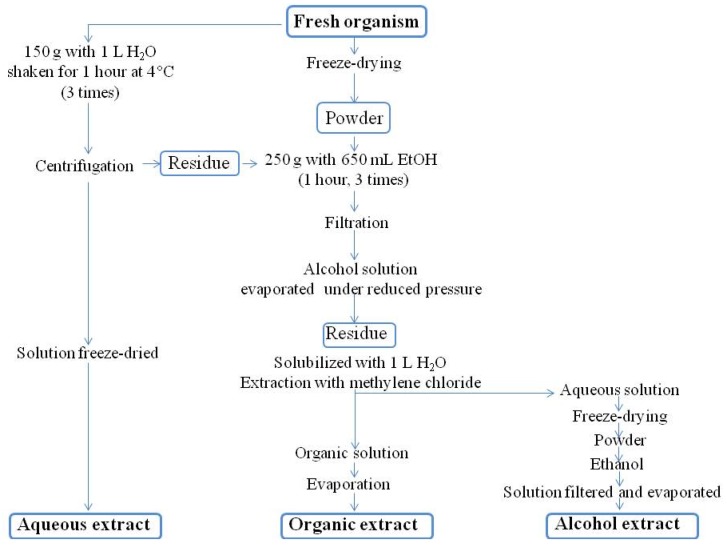
Routine extraction protocol (IRD Nouméa).

Unless otherwise specified, a standard protocol is used for extracting compounds and separating them into crude non-polar (organic) and polar (aqueous) fractions (Figure 4).

Organic fractions are generally evaporated under vacuum (Rotovap) and kept in the dark at −20 °C. Water solubles may need to be desalted using size-exclusion gel chromatography (SEC) or by membrane filtration under nitrogen (Amicon^®^, Alsace, France) prior to separation, but for long-term storage, desalting is not necessary. Aliquoting is necessary for multiple chemical characterizations and for bioassays.

#### 3.3.2. Peptide Protease Inhibitors

Live tissues are processed (minced or fragmented) on board and placed in an acidic solution (methanol/2 N acetic acid) to optimize the extraction of polar substances and block any potential hydrolysis by proteolytic enzymes. The cold (−20 °C) slurry is filtered twice and filtrates are reduced by evaporation and neutralized to pH 5.5 prior to freeze-drying. The total protein content of the crude extract is assayed by measuring optical densities with a UV spectrophotometer.

### 3.4. Separation, Purification

Classical separation and purification techniques (TLC, HPLC, SEC) were not routinely used unless requested for products presenting an interesting bioactivity profile. This data is mentioned in the original publications for each novel structure.

### 3.5. Structural Analysis

Basic spectroscopic (NMR, UV, IR) and spectrometric (MS) methods are used when available on site, mostly to avoid replication.

### 3.6. Chemical Databases

The IRD proprietary information system Cantharella [25] compiles pharmacochemical data of all organisms collected in New Caledonia and cross-Pacific oceanographic cruises for the study of their natural substances, with restricted access via Internet. The information system provides access to biological data, accounts for all chemical processes from extraction to purification, and presents the bioactivity profile detected using the complete range of tests.

## 4. Biological Activities

### 4.1. Preliminary Testing

Vacuum-dried crude extracts or non-purified fractions thereof can be used for describing general characteristics using toxicity assays on various invertebrate, vertebrate and plant models, and cultures of microbial reference strains (Table 1).

Preliminary tests performed on site are useful for making appropriate decisions as to whether a given sample from a newly collected organism is worth investigating further. Along with taxonomic criteria and dereplication issues, such decisions are now taken after consulting the scientific literature and specialized databases, especially the IRD databases LagPlon [24] and Cantharella [25], which, in addition to the more traditional MarinLit and other databases, considerably aid local researchers.

#### 4.1.1. Brine Shrimp Toxicity Assay

The brine shrimp (*Artemia salina*) toxicity bioassay is one of the most basic and widely used tests to detect cytotoxicity. Newly hatched brine shrimp nauplii are exposed to various concentrations of soluble or solubilized test substances and mortality is recorded, leading to a median lethal dose (LD_50_) estimate. A typical protocol is detailed in [27].

#### 4.1.2. Mosquito Fish Toxicity Assay

The mosquito fish *Gambusia affinis* is often used to evaluate the toxicity of substances to be screened for antimitotic activities, and it has been adapted from [28], a study that used this species for comparative ecotoxicological studies of soft corals. The mosquito fish toxicity test has been extended to include multiparametric behavioral observations and can provide valuable neurophysiological information as well as ecotoxicological data on crude extracts that are water soluble or solubilized [29].

#### 4.1.3. Fertilization of Sea Urchin Eggs

The aim of this test is to observe the division pattern of eggs of the test sea urchin *Echinometra mathaei* fertilized in the laboratory, by adapting the method Kobayashi [30] originally designed for testing the toxicity of heavy metals on early embryonic stages. This test is highly sensitive and works at extremely low concentrations of the test substances. Cell division arrest at specific early and embryonic stages provides a preliminary indication of specific enzymatic inhibition at determined steps of the cell cycle.

#### 4.1.4. Anti-Serpin Activity

Serine protease inhibition assays are conducted locally using bovine trypsin (protocol inspired from Green *et al.*, 1953) [36] and porcine elastase [37], with respectively, benzoyl-arginine ethyl ester (BAEE) and succinyl (Alanyl)_3_ para-nitro anilide (Suc [Ala]_3_ pNa) as substrates. End-point titration using pH-stat benchtop equipment is used to evaluate the inhibition potential of the crude extracts on bovine trypsin. The colorimetric method is used to evaluate the anti-elastase activity of the crude extracts.

### 4.2. Further Biological Testing

For the identification of antiproliferative natural products, cancerous and non-cancerous mammalian cell lines are tested. The comprehensive list of cell lines that are used to characterize active molecules from sponges is given in the footnote of Table 2.

Following the preliminary anti-serpin tests (see Section 4.1.4.), other serine proteases (chymotrypsin, subtilisin), cysteine proteases (papain, viral proteinases), aspartic proteases (pepsin, renin) and metalloproteases (thermolysin, carboxypeptidase A) have been carried out by M.A. Coletti-Previero, Montpellier (mainland France).

## 5. Natural Products by Taxon

### 5.1. Porifera (Sponges)

#### 5.1.1. General Comments

Sponges are multicellular, filter-feeding diploblastic invertebrates, *i.e.*, without organized tissues or organs, but with a special inner layer of ciliated cells called choanocytes that generate constant water flow through numerous feeding/excretory channels. There are two major subphyla of Porifera based on their biomineralization patterns: the Calcarea or calcareous sponges and the Silicispongia or siliceous sponges, the latter group being further subdivided into Hexactinellida (glass sponges), Demospongiae, which contain a proteinaceous matrix (spongin), and Homoscleromorpha, now recognized as distinct from the latter class. Calcareous sponges are primarily found in shallow waters, and particularly abundant and diverse in tropical coral reefs. Siliceous sponges, which represent most of the living sponge species today, are found at all bathymetric levels and also in freshwater, with large populations in some rivers and lakes. Most glass sponges live in deep waters, and are difficult to access [16]. Since the 1980s, deep-water dredging on the outer reef slope of the barrier reef down to 800 m has led to the discovery of a number of non-described species. As of 2007, 149 species of Porifera have been recorded in New Caledonian waters [5].

Most shallow-water sponge species live in symbiosis with Archaea, eubacteria and cyanobacteria, forming holobiont systems that represent the main source of bioactive compounds [38,39] primarily isolated from host tissues, but occasionally isolated from cultures of associated microbiota. Sponges contain a wide range of so-called secondary metabolites, some of which afford interesting biological activities [40] (Table 2).

#### 5.1.2. Porifera Success Stories

New Caledonian sponges have revealed more exciting chemicals than any other studied taxon, in terms of carbon skeleton, degree and patterns of unsaturation, halogenation, functional group originality, presence of unusual heteroatoms *etc.* Some of these features are responsible for the rather exceptional bioactivity profiles encountered. Some outstanding examples are described below:

##### *Cymbastela* *Cantharella*

**Figure 5:**
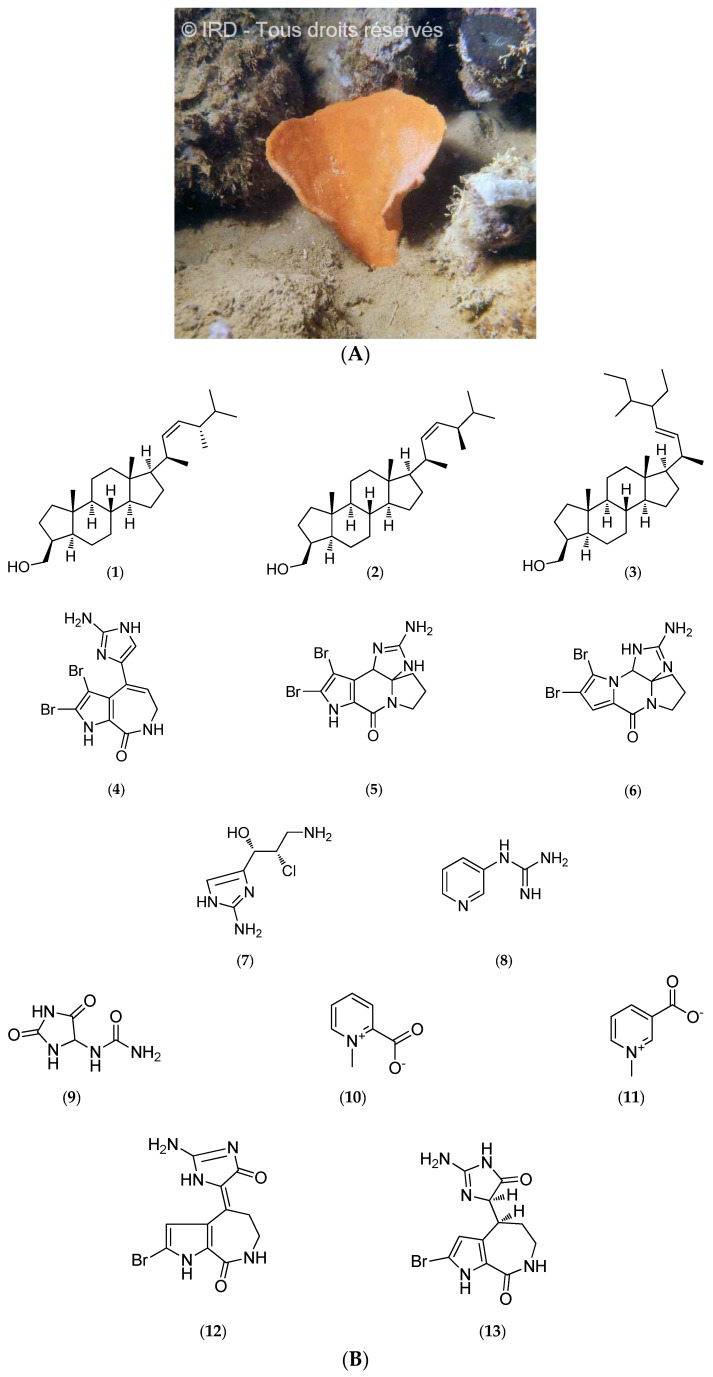
(**A**) Cymbastela cantharella. © IRD; (**B**) Structure of hydroxymethyl-3β-methyl-24S-nor-A-cholest-5α-ene-22-Z (**1**), hydroxymethyl-3β-methyl-24R-nor-A-cholest-5α-ene-22-Z (**2**), hydroxymethyl-3β-ethyl-24ξ-methyl-26ξ-nor-A-cholest-5α-ene-22-E (**3**), odiline (**4**), dibromocantharelline (**5**), dibromophakellin (**6**), girolline (**7**), pyraxinine (**8**), allantoin (**9**), homarine (**10**), trigonelline (**11**), hymenialdisine (**12**) and dihydrohymenialdisine (**13**).

*Cymbastela cantharella* (Family Axinellidae, Class Demospongiae, formerly *Pseudaxinyssa cantharella*, Figure 5A) was collected on the outer south reef of New Caledonia at depths ranging from 10 to 40 m (voucher #R1279, IRD Nouméa).

Chemical analyses led to the isolation of sterols that contain a 3β-hydroxymethyl-A-norcholestane skeleton, a typical feature of the Axinellidae (**1**–**3**, Figure 5B) [100], along with alkaloids such as odiline (**4**, Figure 5B), dibromocantharelline (**5**, Figure 5B) and dibromophakellin (**6**, Figure 5B) [53]. Studies performed on the crude ethanol extracts of *C. cantharella* led to the isolation of girolline (**7**, Figure 5B). Its absolute configuration has been established by X-ray diffraction [56] and total synthesis was achieved few years later [53,57,58,63,100].

Girolline (**7**, Figure 5B) (or girodazole) has demonstrated potent antiproliferative activity *in vitro* on several cell lines such as P388 murine leukemia and P388/dox, a sub-line resistant to doxorubicin, an anthracycline, or KB naso-pharyngeal and T24 bladder carcinoma human cell lines. The half maximal inhibitory concentration (IC_50_) values are 0.06 µM, 0.08 µM, 0.21 µM and 0.19 µM, respectively [55]. Subsequent *in vivo* assays have been performed on several grafted murine tumors used for preclinical evaluation including P388 and L1210 leukemia, and solid tumors such as M5076 histiocytosarcoma and MA/16C mammary adenocarcinoma cells [54,59,63]. Moreover, girodazole (**7**, Figure 5B) also has antitumor activity *in vivo* on the P388/dox cell line. This compound may inhibit the termination step of protein synthesis *in vivo* [60,61]. Although toxicological studies in mice and dogs do not show any major toxic effects, girodazole (**7**, Figure 5B) clinical development was interrupted in 1991 in phase II due to severe side effects, in particular cardiovascular toxicity [59].

Girolline (**7**, Figure 5B) has also shown promising results for the treatment of malaria. This compound has demonstrated potent antiplasmodial activities against four *Plasmodium falciparum* strains with IC_50_ values ranging from 77 to 215 nM, and may act synergistically with chloroquine *in vitro* by affecting protein synthesis [62].

Other studies have led to the characterization of pyraxinine (**8**, Figure 5B), a novel nitrogenous compound, along with previously identified allantoin (**9**, Figure 5B), homarine (**10**, Figure 5B) and trigonelline nitrogen compounds (**11**, Figure 5B) [63], hymenialdisine (previously identified; **12**, Figure 5B) and new pyrrole-2-aminoimidazole alkaloids, which are dihydrohymenialdisine derivatives (**13**, Figure 5B) [64]. Further investigations revealed that hymenialdisine (**12**, Figure 5B) is a powerful inhibitor of cyclin-dependent kinases such as CDK1/cyclin B, CDK2/cyclin A and CDK2/cyclin E (IC_50_ values of 22 nM, 70 nM, and 40 nM, respectively), CDK5/p25 (IC_50_ of 28 nM), as well as against glycogen synthase kinase-3β (GSK-3β) with an IC_50_ of 10 nM and casein kinase 1 (CK1) with an IC_50_ of 35 nM [44].

##### *Echinochalina* *Bargibanti*

**Figure 6:**
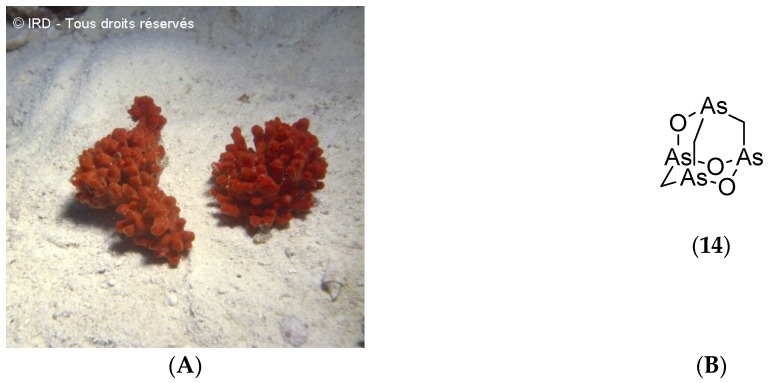
(**A**) *Echinochalina bargibanti*. © IRD; (**B**) Structure of arsenicin A (**14**).

The demosponge *Echinochalina bargibanti* (voucher #R1858, IRD Nouméa, Figure 6A) was collected along the northeastern coast of Grande Terre between 18 and 25 m depth during the SMIB program. A bioassay-guided fractionation of its organic extracts led to the isolation of arsenicin A (**14**, Figure 6B), the first polyarsenic compound ever found in nature.

Arsenicin A (**14**, Figure 6B) demonstrates potent bactericidal and fungicidal activities against human pathogenic strains such as *Staphylococcus aureus*, *Escherichia coli* and *Candida albicans* with inhibition circles of 24, 28 and 26 mm respectively at 10 µg per disk. In contrast, gentamicin, which is the reference antibiotic, induces inhibition circles of 22, 30 and 22 mm [68]. Arsenicin A (**14**, Figure 6B) has been synthesized and its crystal structure determined [101]. An improvement in this synthesis has led to (±)-arsenicin A which shows potent antiproliferative activity on acute promyelocytic leukemia cell lines. This compound is more potent than arsenic trioxide (Trisenox) which is used for treating acute promyelocytic leukemia. (±)-Arsenicin A has also demonstrated antiproliferative activity against pancreatic adenocarcinomas and glioblastomas [102].

##### *Dendrilla* sp.

*Dendrilla* sp. (voucher #R171, IRD Nouméa) is a common shallow-water (lagoon, at approximately 20 m depth) dendroceratid sponge belonging to the Demospongiae. It features an unusual “scouring pad” appearance with its spongin fibers protruding from an elastic tissue mass that can vary in color from dark green to reddish.

**Figure 7:**
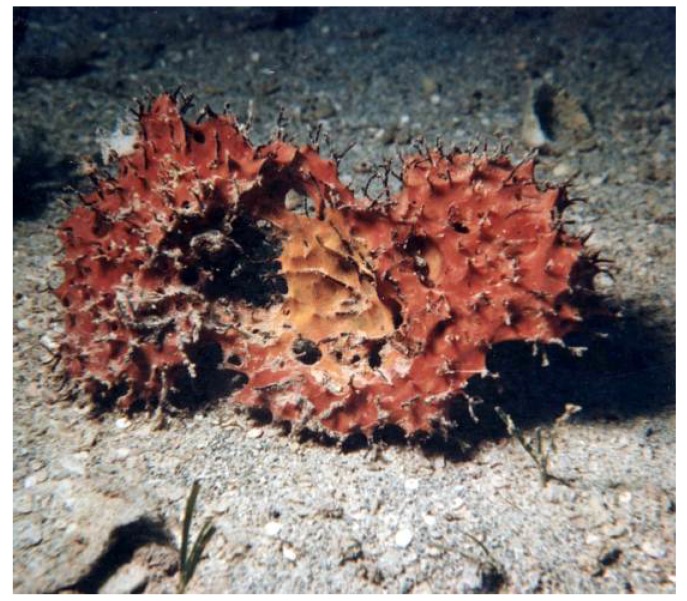
*Dendrilla nigra*. Photo: Philippe Plailly (CNRS).

R171 is cytotoxic in brine shrimp larvae bioassays [103], and its crude aqueous extract is toxic to the mosquito fish *Gambusia affinis*, causing erratic swimming patterns followed by 100% mortality within 12 h [29]. Reported biological activities of the crude organic extracts of *Dendrilla nigra* (Figure 7) include antitumor, anti-inflammatory and antimicrobial [103] properties with potential interest for shrimp aquaculture for treating *Vibrio* pathogens [104].

**Figure 8:**
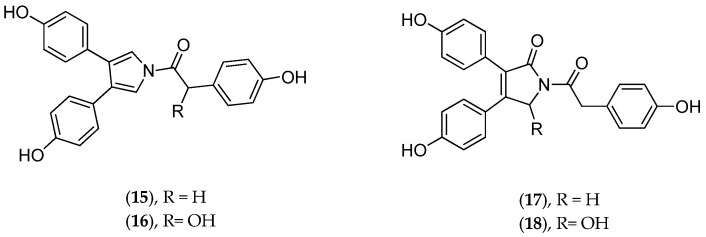
Structure of neolamellarin A (**15**), 7-hydroxylamellarin A (**16**), neolamellarin B (**17**) and 5-hydroxylamellarin B (**18**).

Chemically, dendrillid sponges are known to contain lamellarins A–B (**15**–**18**, Figure 8), aromatic alkaloids of probable symbiotic origin. These pyrrole derivatives are antitumoral (HIF-1 inhibitors) [105]. *Dendrilla cactos* contains cyclic bastadins, which are bromotyrosine-derived peptides endowed with Gram+ antibacterial and anti-inflammatory properties; in addition they inhibit topoisomerase II, dehydrofolate reductase and the endothelin A receptor [106]. Cold-water *Dendrilla membranosa* contains membranolides used as natural antifeedants and other microbe-derived bioactive compounds [107]. The suspicion that indwelling bacteria may be responsible for much of the reported bioactivities in sponges in the *Dendrilla* genus has prompted the taxonomic characterization of culturable strains in *Dendrilla nigra* [108].

Another interest in *Dendrilla* R171 stems from bioassays as serine protease inhibitors (§ 4.1.4) with remarkable anti-trypsin and anti-elastase activities in preliminary benchtop assays on crude extracts. With up to 100% inhibition on both bovine trypsin and porcine elastase activities, this sponge displays the most potent anti-serpin activity of the 133 invertebrate species tested in total. In 1988, collaborators in Montpellier isolated the active compound (a peptide) from the water-ethanol extract, bioguided by an anti-elastase assay (porcine and human) and they sequenced a 43-residue amino acid, but the structure was never published. This was long before aeruginosins, peptide anti-serine proteases first isolated from cyanobacteria in 1994. A whole family of more than 20 aeruginosins has been described since then, some from sponges of the genus *Dysidea* [109]. The presence of such compounds may explain the anti-trypsin activity if aeruginosins are also present in *Dendrilla* R171 (which has not been verified), but anti-elastase activity has never been associated with these molecules.

Thus, the identity of the trypsin and elastase inhibitor(s) from *Dendrilla* R171 remains unknown (or at least unpublished) to date. The fact that another tested *Dendrilla* (voucher #1225) does not display any inhibitory activity suggests that microbial symbionts—possibly cyanobacteria—are involved in the reported R171 activities.

##### *Niphates* sp.

**Figure 9:**
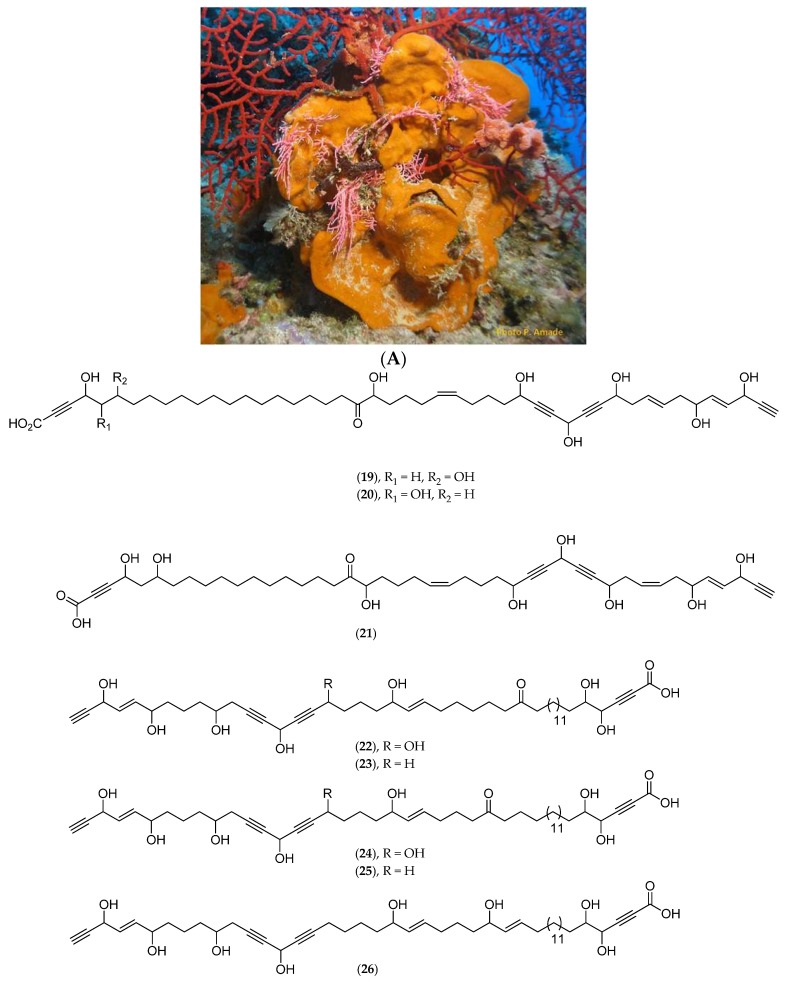

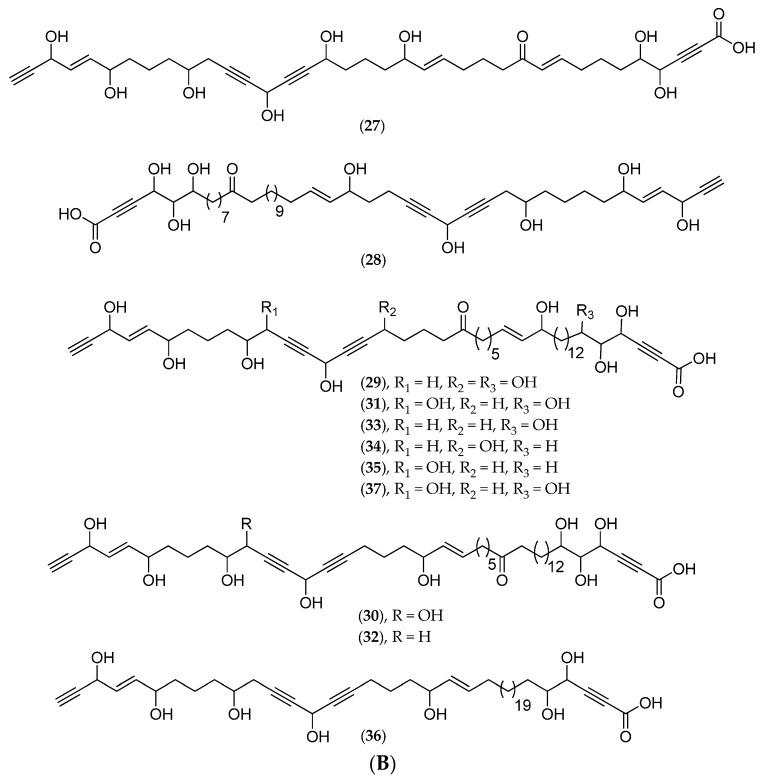
(**A**) *Niphates* sp. Photo: Philippe Amade (INSERM); (**B**) Structure of nepheliosyne A (**19**), nepheliosyne B (**20**), petrosolic acid (**21**), osirisynes A–F (**22**–**27**), haliclonyne (**28**) and fulvynes A–I (**29**–**37**).

A new C_47_ polyoxygenated acetylenic acid, nepheliosyne B (**20**, Figure 9B), along with the previously described nepheliosyne A (**19**, Figure 9B), have been isolated from *Niphates* sp. (Figure 9A), collected in 2008 in a southwest lagoon at 22 m depth (voucher #MHNM 1646, Natural History Museum of Marseille, mainland France). Both nepheliosyne A and B have shown cytotoxicity against K562 chronic myelogenous leukemia, U266 myeloma, SKM1 myelodysplastic syndrom and Kasumi acute myeloid leukemia human cell lines with IC_50_ values ranging from 150 to 200 µM [86]. Several polyhydroxylated acetylenic metabolites of marine sponges with a diacetylenic carbinol and a α-yne carboxylic group have been reported: nepheliosyne A (**19**, Figure 9B) from *Xestospongia* sp., petrosolic acid (**21**, Figure 9B) from *Petrosia* sp., osirisynes (**22**–**27**, Figure 9B) and haliclonyne (**28**, Figure 9B) from *Haliclona* sp., and fulvynes A–I (**29**–**37**, Figure 9B) from *Haliclona* *fulva*. Nepheliosyne B (**20**, Figure 9B) is, along with nepheliosyne A (**19**, Figure 9B) and petrosolic acid (**21**, Figure 9B), the third example of a linear acetylene with diacetylenic carbinol, α-hydroxyketone, and α-yne carboxylic groups. All these data suggest that, from a chemotaxonomic point of view, polyhydroxylated acetylenic metabolites constitute potential markers of Haplosclerida species.

##### *Corallistes* sp.

**Figure 10:**
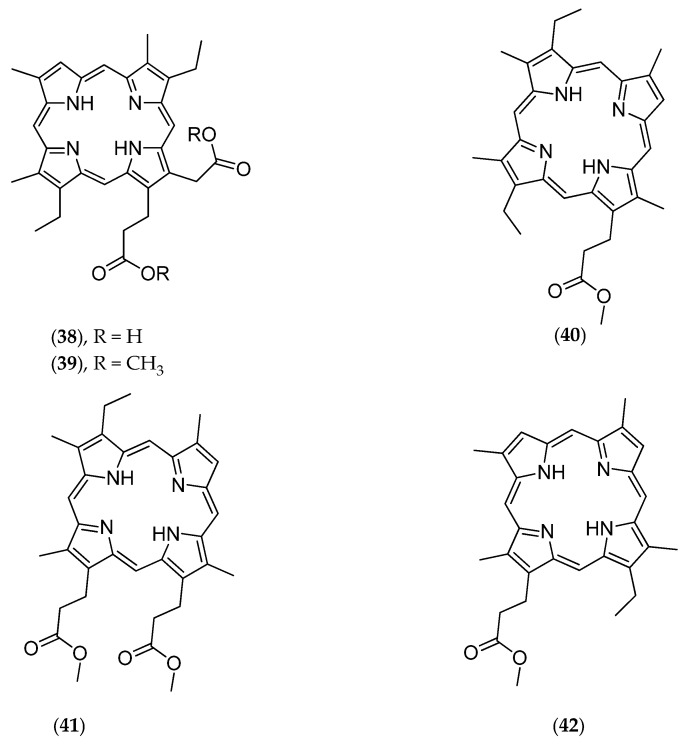
Structure of corallistin methyl ester A (**38**), B (**39**), C (**40**), D (**41**), and E (**42**).

This deep-sea sponge was collected by beam trawl at a depth of 350 m in the Coral Sea. The crude dichloromethanol extract shows cytotoxic activities against the KB naso-pharyngeal carcinoma cell line with an IC_50_ of 10 µg/mL. Subsequent chemical analyses led to the isolation of a free porphyrin called corallistin A (**38**, Figure 10) [50]. Its total synthesis followed a few years later [110].

Further studies conducted on *Corallistes* sp. led to the isolation of corallistins B (**39**, Figure 10), C (**40**, Figure 10), D (**41**, Figure 10) and E (**42**, Figure 10). These porphyrins may be used as new photosensitizers in phototherapy, particularly by generating singlet oxygen toxic to cancer cells [51].

### 5.2. Ascidians

#### 5.2.1. General Comments

Ascidians are invertebrate filter feeders that belong to phylum Chordata. They represent the most evolved invertebrates with a heart and a respiratory system. Living in both solitary and colonial sessile modes, ascidians can be found in all the world’s seas and at all depths. The external tunic of sea-squirts (tunicates) is made of tunicin, a cellulosic substance that is extremely rare in the animal kingdom [19]. Didemnids are soft-bodied, bag-like organisms. Like sponges, didemnids can harbor cyanobacterial photosymbionts, e.g., the photosynthetic genus *Prochloron* which lives in symbiosis with its host [111].

This class has a special “defense” feature: they can concentrate some toxic elements including heavy metals (notably vanadium), hydrocarbons and elementary sulfur, which may be found in *Polycarpa aurata,* or even sulfuric acid concentrated inside tiny vesicles of the outer tunic. Studies have also revealed high amounts of nitrogenous compounds (cyclic peptides and alkaloids) with powerful biological activities [112] (Table 3). As of 2007, 290 tunicates had been identified in New Caledonian waters [5].

#### 5.2.2. *Lissoclinum bistratum*

**Figure 11:**
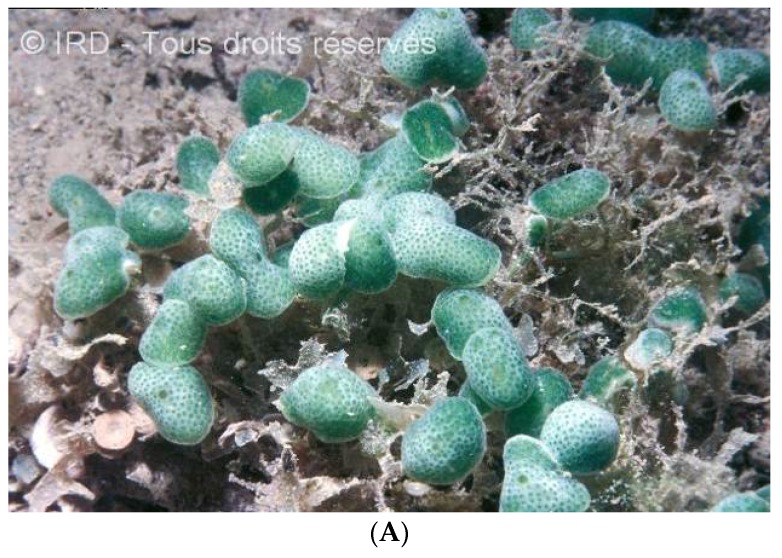

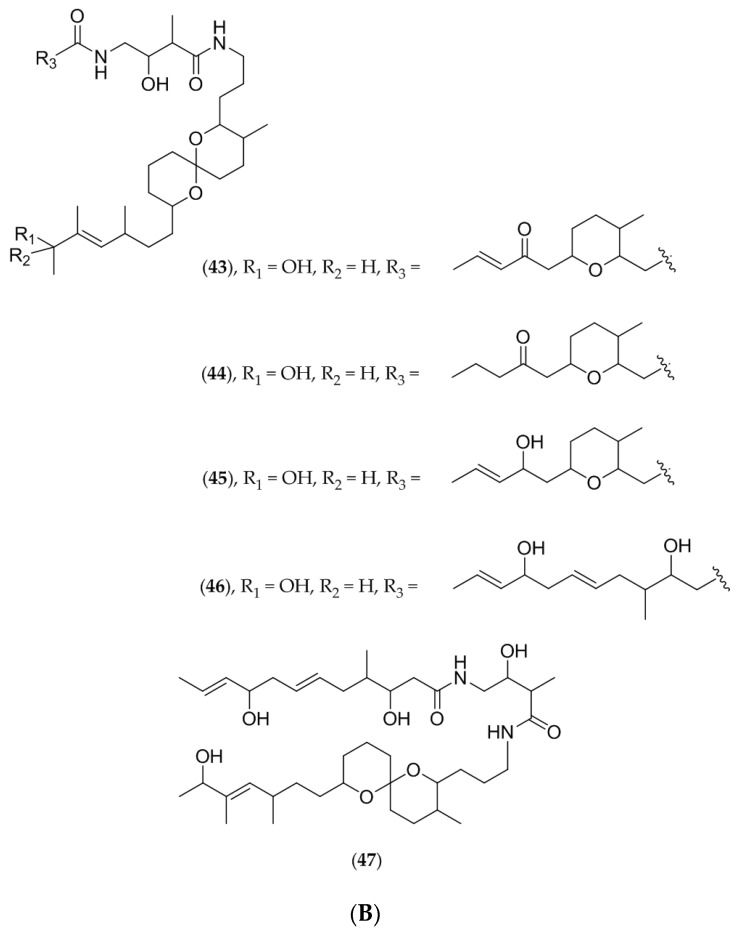
(**A**) *Lissoclinum bistratum*, © IRD; (**B**) Structure of bistramide A (**43**), B (**44**), C (**45**), D (**46**), and K (**47**).

The didemnid *Lissoclinum bistratum* (voucher # UA264, IRD Nouméa, Figure 11A) was collected near the Ua islet in the southwest lagoon. Chemical studies followed when two cases of human intoxication were observed during manipulation of the lyophilized powder. The crude dichloromethanol extract of *L. bistratum* demonstrates acute toxicity on rats, mice and rabbits. Moreover, it has revealed cytotoxic activities with IC_50_ values of 45 nM, 20 nM and 22 nM on KB, P388 and normal human endothelial cells, respectively [115].

Separation and purification led to the isolation of a tetrahydropyran derivative called bistramide A (**43**, Figure 11B) (or bistratene). Its partial structure has been characterized using two dimensional NMR techniques [125].

Bistramide A (**43**, Figure 11B) appears to block the division of NSCLC-N6 (L16) cells at the G1 phase after a 24 h incubation period at a concentration of 1.42 µM. The IC_50_ value is 0.49 µM. Bistramide A (**43**, Figure 11B) may also induce polyploidy, causing unsuccessful cytokinesis [116]. It has revealed immunomodulator activities (suppressor/stimulator) on the proliferation of T and B cells [120]. Moreover, demonstrated using the voltage-clamp technique, bistramide A (**43**, Figure 11B) has proven to be responsible for a resting block by inhibiting sodium channels [118] and can bind to contractile proteins in competition with calcium in frog skeletal muscle fibers [119]. 

Later investigations performed on *L. bistratum* led to the isolation of new tetrahydropyran derivatives called bistramides B (**44**, Figure 11B), C (**45**, Figure 11B), D (**46**, Figure 11B) and K (**47**, Figure 11B). Bistramides A (**43**, Figure 11B), B (**44**, Figure 11B) and C (**45**, Figure 11B) show potent cytotoxic activities with IC_50_ values of less than 1 µg/mL on several cancer cell lines including KB, P388, P388 doxorubicin-resistant, B16, HT29, NSCLC-N6, MRC5CV1 fibroblasts and T24 bladder carcinoma cells. Bistramide K (**47**, Figure 11B), which is the least toxic compound, induces a complete blockage of NSCLC-N6 cells in the G1 phase after a 48 h incubation period with an IC_50_ value of 3.23 µg/mL [117,126]. 

### 5.3. Cnidaria

This phylum is divided into two subclasses, Octocorallia, which includes alcyonarians, gorgonians and pennatularians, and Hexacorallia, which includes scleratinarians. The chemical and biological properties of Cnidaria collected in New Caledonia are reported in Table 4.

#### 5.3.1. Alcyonarians

##### General Comments

Alcyonarians also called “soft corals” because they lack a calcium carbonate skeleton. This carbonate skeleton is replaced in most species by aragonitic sclerites, which are of major taxonomic importance for identification. They are benthic, sessile, colonial organisms often composed of many individual polyps stemming from a common sterile trunk, which is attached to the substratum by a glycoprotein “glue”. The main families are the Alcyoniidae, Nephtheidae and Xeniidae.

Alcyonarians often possess endodermal zooxanthellae which provide them with oxygen and photosynthates due to their photosynthetic activity. A total of 173 species have been reported in New Caledonian waters [5].

“Cocktails” of toxic secondary metabolites, especially diterpenes, occur in their tissues, and serve anti-predator (toxic and emetic effects) [130] and anti-competitor defense functions (contact necrosis and allelopathic growth inhibition [131], but also as anti-fouling substances and as egg protectants [132]).

Octocorals, which includes alcyonarians, gorgonians and pennatularians, are chemically characterized by terpenes, oxylipins such as prostaglandins and prostanoids, sterols and some aromatic derivatives [133]. These compounds have promising pharmacological value (Table 4).

##### *Xenia* *Garciae*

**Figure 12:**
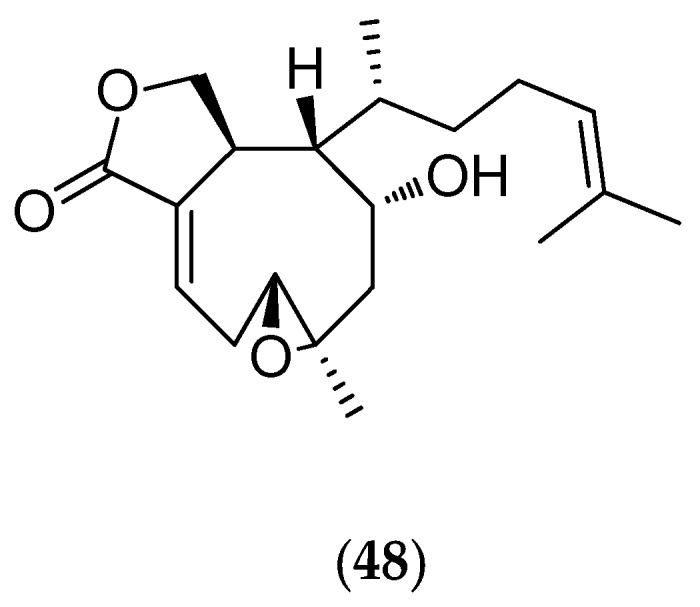
Structure of xenicane (**48**).

The “soft coral” *Xenia garciae* (voucher #3526, Northern Territory Museum, Darwin, Australia) was collected in shallow-water fringing reefs at a depth less than five meters. Chemical analyses isolated an original xenicane diterpene (**48**, Figure 12) that inhibits the growth of *Ceramium codii*, which is a common benthic Rhodophyta contributing to the process known as “fouling” [33]. Around the xenicane skeleton, as around the cembrane skeleton of alcyonids, are found dozens of bioactive diterpenes. The xenicane diterpenes, also found in some brown algae (Dictyotales), are particularly interesting as synthetic templates for the design of antitumor drugs [134].

#### 5.3.2. Gorgonians

##### General Comments

Also known as sea whips or sea fans, gorgonians are “animal-flowers” that are supported either by a calcareous skeleton (suborder Scleraxonia) or by a flexible horny skeleton (suborder Holaxonia) made of a fibrous protein, gorgonin. Exclusively marine, these sessile invertebrates can live as solitary animals or in colonies. They are found in many places in all oceans and can occur in different growth forms such as bushes, whip-like branches and sometimes as blunt lobes or even flat crusts [18]. As of 2007, 93 species of gorgonians have been described in New Caledonian waters [5].

##### *Melithea* cf. *Stormii*

**Figure 13:**
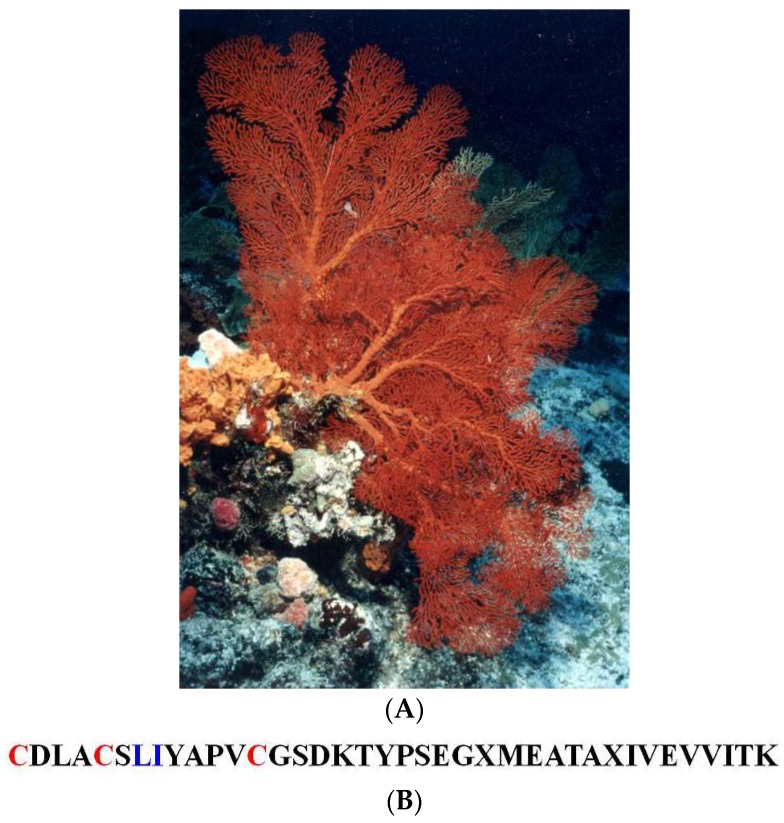
(**A**) *Melithea cf. stormii*. Photo: Philippe Plailly (CNRS); (**B**) *N*-terminal amino acid sequence of *iela melst* (**49**). Half cysteine residues are noted in red, and correspond to half-cysteines of the non-classical Kazal-type inhibitor. The blue pair indicates P1-P1’ residues around the putative active site. One-letter codes for amino acids: A, alanine; C, cysteine; D, aspartate; E, glutamate; G, glycine; I, isoleucine; K, lysine; L, leucine; M, methionine; P, proline; S, serine; T, threonine; V, valine; X, unknown; Y, tyrosine.

The sea fan *Melithea* cf. *stormii* (Figure 13A) is a large scleraxonian gorgonian commonly found on outer reef slopes. The voucher sample and the biological material used for sequencing and bioassays were taken from only one medium-sized specimen (voucher #HG163, IRD Nouméa) collected in Uitoe Pass, Southern Province at 20 m depth. Below, we summarize the main highlights of this specimen HG163; all details can be found in [37].

From HG163, a 95% pure novel peptide elastase inhibitor was isolated using SEC and reversed-phase chromatography. This peptide of 39 residues in length was hereafter referenced as *iela melst* (**49**, Figure 13B) according to nomenclature. The amino acid sequence of the first 20 *N*-terminal residues of *iela melst* (**49**, Figure 13B) is homologous to that of *iela anesu* from the sea anemone *Anemonia sulcata*, a non-typical Kazal-type elastase inhibitor [135]. The Cha procedure [136] was used to monitor the inhibition kinetics of the amidolysis of the synthetic substrate [Suc(Ala)_3_pNA] by porcine pancreatic elastase (PPE) at different concentrations using *iela melst* (**49**, Figure 13B). This inhibitor works as a slow, tight-binding inhibitor of PPE. The equilibrium dissociation constant K_i_ (1.5 nM) of the complex is quite similar to that of the PPE-elafin complex, the latter being an antileuko-proteinase isolated from patients with psoriasis. The anti-elastase activity of *iela melst* (**49**, Figure 13B) is very comparable to those of most natural or synthetic PPE inhibitors. The presence of such a strong anti-elastase substance in gorgonian cortical tissues may hinder various necrotic processes caused by epibiotic fouling or extracoelenteric digestion by other coelenterates [37].

##### *Villogorgia* *Rubra*

**Figure 14:**
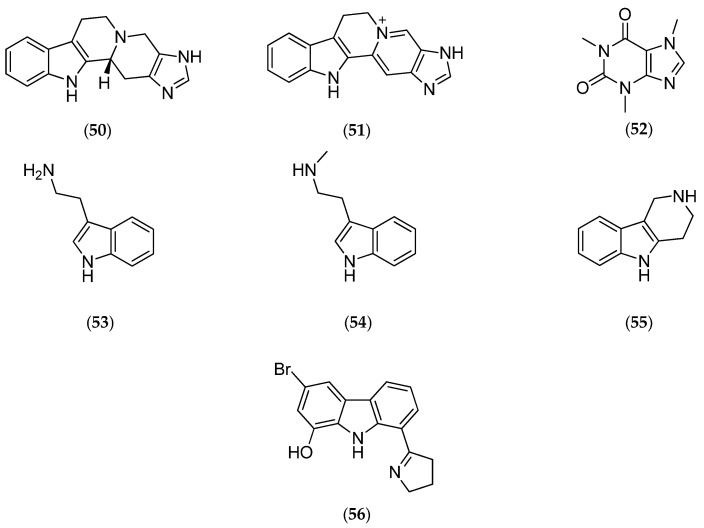
Structure of villogorgin A (**50**), villogorgin B (**51**), caffeine (**52**), tryptamine (**53**), *Nb*-methyltryptamine (**54**), 1,2,3,4-tetrahydro-β-carboline (**55**) and eudistomidin-A (**56**).

Studies performed on the gorgonian *Villogorgia rubra,* collected near the Chesterfield Islands, led to the characterization of two new alkaloids called villogorgin A (**50**, Figure 14) and B (**51**, Figure 14), along with known compounds: caffeine (**52**, Figure 14), tryptamine (**53**, Figure 14), *Nb*-methyltryptamine (**54**, Figure 14) and 1,2,3,4-tetrahydro-β-carboline (**55**, Figure 14).

Villogorgin A (**50**, Figure 14) inhibits (i) the contraction of the guinea pig ileum induced by acetylcholine; (ii) the aggregation of human platelets induced by thrombin and calcium ionophore A23187; this inhibition may be due to modulation of calcium/calmodulin-dependent enzymes. Villogorgin A (**50**, Figure 14) and B (**51**, Figure 14) are structurally related to the marine tunicate β-carboline alkaloid eudistomidin-A (**56**, Figure 14), a strong calmodulin antagonist [127].

#### 5.3.3. Pennatularians

##### General Comments

Sea pens (sea feathers)—also called pennatularians—are a specialized and morphologically distinct group of octocorallian cnidarians.

Pennatularians are made of a thin tissue called the coenenchyme. These sessile animals live in colonies that are built from a single large primary polyp, the oozoid. They have been encountered in all oceans down to 6100 m depth [137].

##### *Lituaria* *Australasiae*

**Figure 15:**
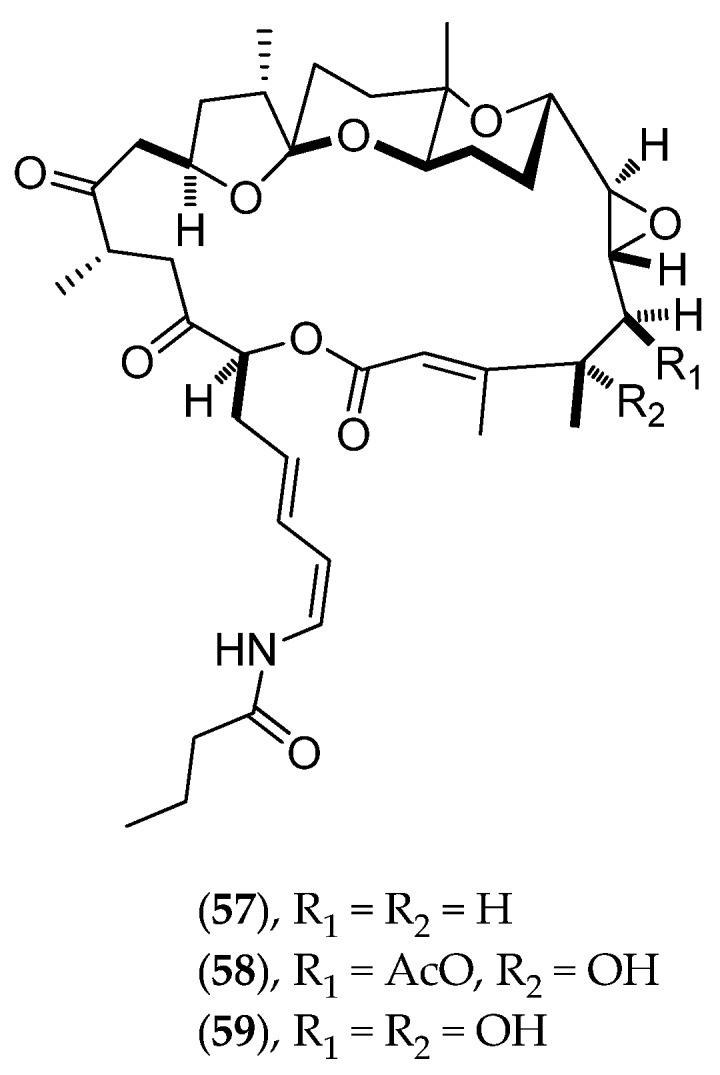
Structure of lituarine A (**57**), lituarine B (**58**) and lituarine C (**59**).

*Lituaria australasiae* was collected at night, inside St. Vincent Bay (west coast, Southern Province). From this octocoral, a novel class of macrocyclic lactones has been identified: lituarines A (**57**, Figure 15), B (**58**, Figure 15) and C (**59**, Figure 15). Lituarines have demonstrated antifungal activities on *Fusarium oxysporum*, *Helminthosporium turscicum*, *Penicillium italicum* and *Phytophtora parasitica*, as well as cytotoxic activities on KB cells with IC_50_ values of 3.7–5.0 ng/mL for lituarine A (**57**, Figure 15), 1–2 ng/mL for lituarine B (**58**, Figure 15) and ranging from 5–6 ng/mL for lituarine C (**59**, Figure 15) [128].

#### 5.3.4. Scleractinians

Scleractinians are also called “stony corals” or “hard corals”. Scleractinian colonies act as biological photosystems during sunlit hours and feed on plankton at night. Closely related to sea anemones, they are also armed with cnidocytes that can be used for predation as well as for territorial defense [138].

“Stony corals” have a large range of colonial growth forms, from crustose to massive, but mostly branching, corymbose, tabular or lamellate, with a few solitary and free-living forms, e.g., mushroom corals [14]. All corals originate from a minute swimming planula that settles and metamorphoses into a single solitary polyp, which itself undergoes successive budding to form a colony that encases itself in an aragonitic (calcium/magnesium carbonate) shell. Growth rates can vary from a few centimeters per year for fast growing branching acroporids that are shorter-lived and rarely very large, to a few millimeters per year at most for large poritid corals that can grow up to 10 m in diameter and live for many centuries.

Shallow-water corals contain *Symbiodinium* (symbiotic unicellular microalgae known as zooxanthellae) within their endodermal cells. These microalgae give the coral its color, which thus can vary in hue depending on the symbiont species. Scleractinians are usually photosymbiotes.

As of 2007, 310 species of scleractinians have been reported in New Caledonian waters [5]. Furthermore, the anthozoan Hexacorallia class may produce toxins (e.g., palytoxin) and other secondary metabolites such as venoms, similar to those of sea anemones, or photoprotection pigments (e.g., mycosporins, zoanthoxanthins) [139].

### 5.4. Echinoderms

#### 5.4.1. General Comments

Phylum Echinodermata is divided into two subphyla, the Pelmatozoa including the class of Crinoidea (crinoids, sea lilies and feather stars), and the Eleutherozoa which comprises Asteroidea (starfish, sea stars), Echinoidea (sea urchins), Holothuroidea (sea cucumbers) and Ophiuroidea (brittle stars) [17].

Echinoderms are exclusively marine invertebrates that have an endoskeleton consisting of magnesium calcite. In all, 257 species of echinoderms have been reported in New Caledonian waters as of 2007 [5].

The therapeutic potential of these marine organisms is outlined in Table 5. Secondary metabolites produced by Crinoidea are sulfated anthraquinonic pigments, whereas Eleutherozoa produce quinonic pigments, naphthoquinones or sulfated saponins (e.g., asterosaponins, holothurins) [140].

#### 5.4.2. *Actinopyga Flammea*

**Figure 16:**
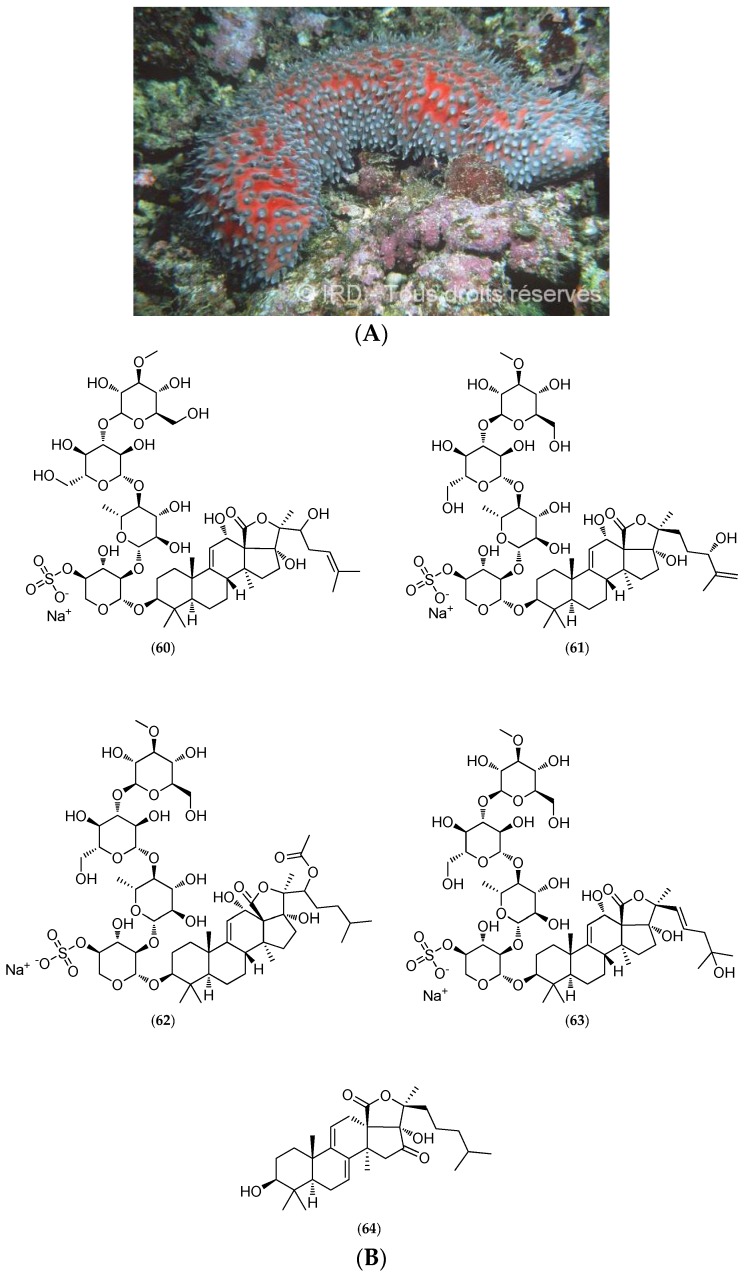
(**A**) *Actinopyga flammea*. © IRD; (**B**) Structure of 24(*S*)-hydroxy-25-dehydro-echinoside A (**60**), 22-hydroxy-24-dehydro-echinoside A (**61**), 22-acetoxy-echinoside A (**62**), 25-hydroxy-dehydroechinoside A (**63**) and 16-keto-holothurinogenin (**64**).

The sea cucumber *Actinopyga flammea* (voucher #EH 025, IRD Nouméa) was collected at depths ranging from 35 to 50 m on the outer reef slope on the New Caledonian west coast. Highly ichthyotoxic, this species is used by fishermen in the Philippines to kill fish in coral holes. A specimen similar to that shown in Figure 16A reportedly caused the death of all fish overnight after a caretaker accidentally placed it in a fish tank at the Aquarium of Nouméa.

Chemical studies led to the purification and characterization of triterpenoid saponins comprising the novel 24(*S*)-hydroxy-25-dehydro-echinoside A (**60**, Figure 16B), 22 ξ-hydroxy-24-dehydro-echinoside A (**61**, Figure 16B), 22 ξ-acetoxy-echinoside A (**62**, Figure 16B) and 25-hydroxy-dehydroechinoside A (**63**, Figure 16B) which were minor compounds. A new sapogenin, 16-keto-holothurinogenin (**64**, Figure 16B), was also obtained by acid hydrolysis of crude saponins [145].

#### 5.4.3. *Gymnochrinus Richeri*

**Figure 17:**
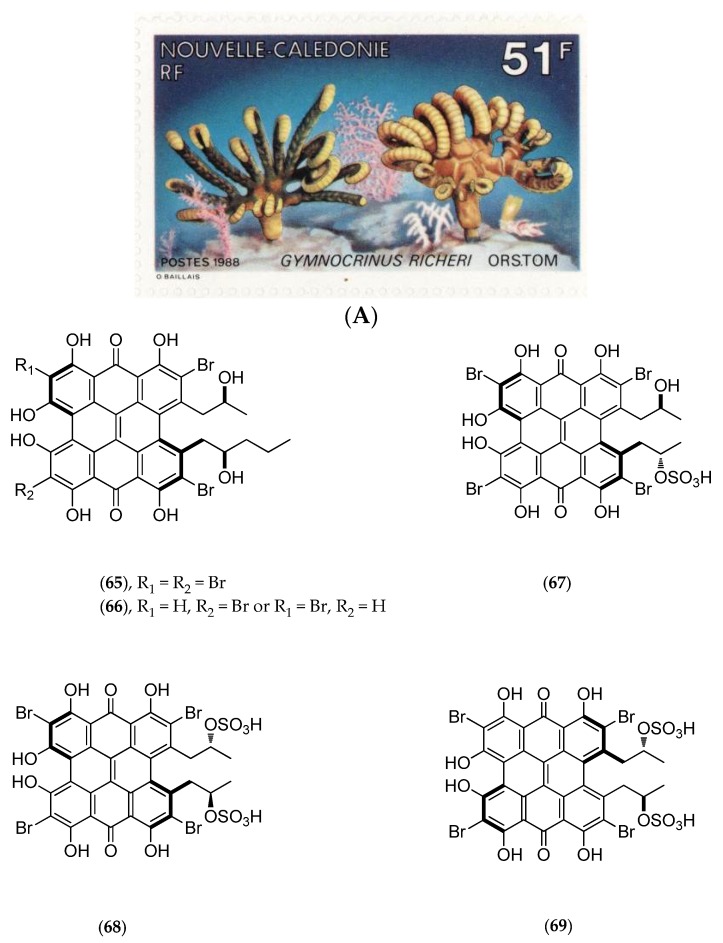

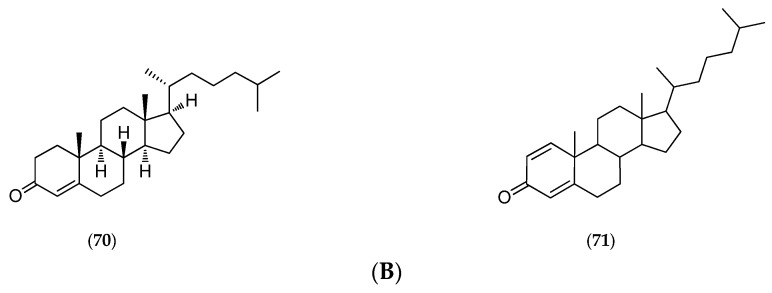
(**A**) *Gymnochrinus richeri.* Postage stamp issued by the *Office des Postes et Télécommunications* **(1988)** (courtesy of a private collection); (**B**) Structure of gymnochrome A (**65**), B (**66**), C (**67**), D (**68**), isogymnochrome D (**69**), cholest-4-en-3-one (**70**) and cholesta-1,4-dien-3-one (**71**).

The “living fossil” *Gymnochrinus richeri* (voucher MNHN, Paris, mainland France, Figure 17A) was collected in 1987 at a depth of 520 m on Stylaster Bank, Norfolk Ridge. Chemical studies isolated gymnochromes A–D (**65**–**68**, Figure 17B), isogymnochrome D (**69**, Figure 17B), which constitute a new group of brominated phenanthroperylenequinones with an hypericin core [141], and also of some sterols such as cholest-4-en-3-one (**70**, Figure 17B) and cholesta-1,4-dien-3-one (**71**, Figure 17B) [146].

Gymnochromes B (**66**, Figure 17B), D (**68**, Figure 17B) and isogymnochrome D (**69**, Figure 17B) possess antiviral activities *in vitro* against the human immunodeficiency virus (A. Bousseau, unpublished results) and the dengue virus with a 50% viral titer reduction factor (RF_50%_) of less than 1 µg/mL [142].

Further studies have revealed that photoexcitation of gymnochrome A (**65**, Figure 17B) may induce electrophysiological effects such as the blockage of background K^+^ current in the atrial region of the frog heart muscle [147]. Moreover, gymnochrome B (**66**, Figure 17B) has demonstrated powerful virucidal and antiviral photoactivity with median effective dose (ED_50_) of 0.042 nM/mL and 0.029 nM/mL, respectively. This photoactivity may be partly due to its brominated hypericin core [148].

#### 5.4.4. *Thromidia Catalai*

**Figure 18:**
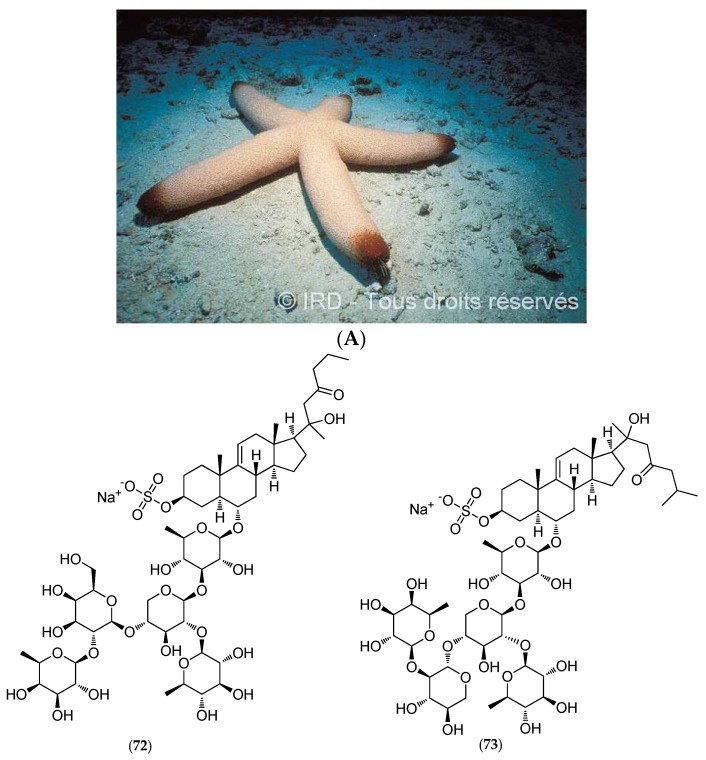

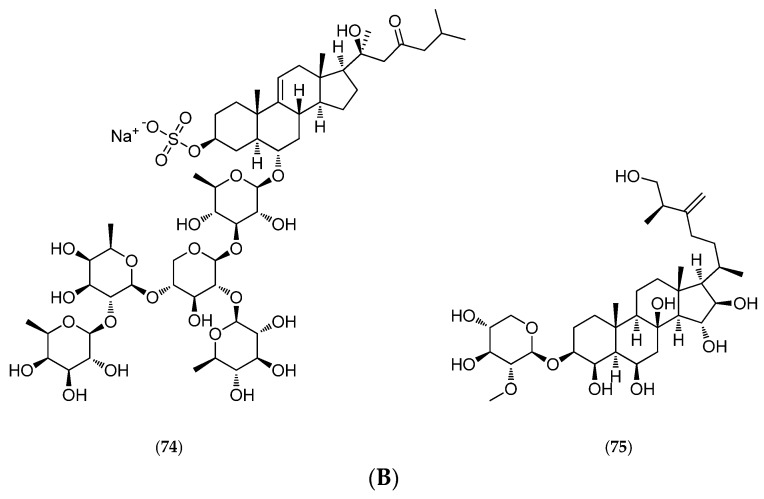
(**A**) *Thromidia catalai*. © IRD; (**B**) Structure of thornasteroside A (**72**), ophidianoside F (**73**), regularoside B (**74**) and thromidioside (**75**).

*Thromidia catalai* (voucher #EA065, IRD Nouméa, Figure 18A) is one of the largest known starfish species, discovered by René Catala in the inner lagoon close to Dumbea Pass in the barrier reef of the Southern Province, around 25 m in depth. This massive five-armed species reaches up to 60 cm in diameter and weighs several kilograms.

In addition to three previously described sulfated asterosaponins (thornasteroside A (**72**, Figure 18B), ophidianoside F (**73**, Figure 18B) and regularoside B (**74**, Figure 18B)), which belong to a family of strong surfactants with ichthyotoxic properties, polar *T. catalai* extracts revealed a new steroid monoglycoside called thromidioside (**75**, Figure 18B) [149].

### 5.5. Macroalgae

Macroalgae belong to three different divisions: red algae (Rhodophyta), brown algae (Heterokontophyta, also known as the Ochrophyta, class Phaeophyceae) and green algae (Chlorophyta). All three evolutionary lineages diverged early on according to their respective plastid configurations, reflecting their endosymbiotic history [150].

Algae have a relatively unspecialized vegetative apparatus called a thallus. Important differences are noted in many ultrastructural and biochemical features including photosynthetic pigments, storage compounds, composition of cell walls, presence/absence of flagella, ultrastructure of mitosis, connections between adjacent cells, and the fine structure of chloroplasts. They vary from small, single-celled forms to complex multicellular forms, such as the giant kelp *Macrocystis pyrifera* that can grow up to 49 m in length in New Zealand and holds the record of the fastest growing organism (0.6 m per day).

Marine macroalgae generally live attached to rocks or other hard substrata in coastal areas but can be also found in sandy areas such as the Udoteaceae in tropical areas. They occur in all of the world’s oceans where they occupy the seabed (phytobenthos), although a few species float freely on the surface (e.g., the pelagic algae of the Sargasso Sea). In New Caledonia [151], the large brown algae are mainly Fucales (*Sargassum*, *Turbinaria*) and Dictyotales and form large stands on rocky lagoon bottoms, while several Chlorophyta species from the genera *Halimeda* and *Caulerpa* colonize sandy bottoms [152,153,154,155]. The large fleshy Rhodophyta are mainly restricted to outer slopes or the cooler waters of the southern lagoon. As of 2007, 454 species of algae and marine angiosperms had been reported in New Caledonian waters [5].

**Figure 19:**
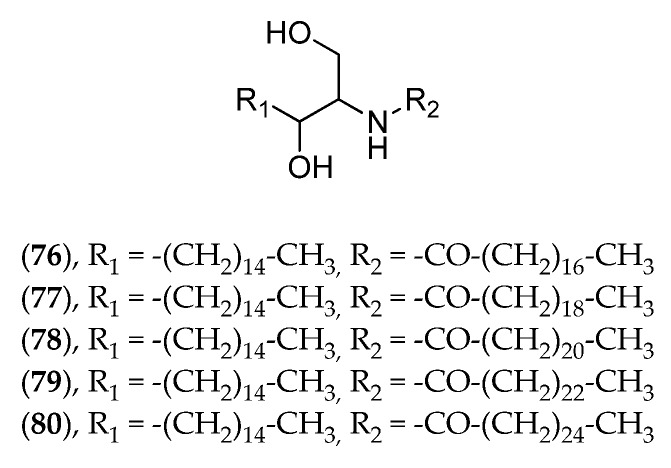
Structure of caulerpicin C18 (**76**), C20 (**77**), C22 (**78**), C24 (**79**) and C26 (**80**).

Despite their capacity to produce large amounts of natural products, macroalgae from New Caledonia have been little documented in this respect. *Caulerpa* species were investigated for caulerpicin (**76**–**80**, Figure 19) activity in the 1980s for cosmetic purposes, but the highly publicized invasion of *Caulerpa taxifolia* in Mediterranean Sea brought the project to an immediate halt [156]. The genus *Lobophora* (Phaeophyta) has been investigated in biological interaction studies between macroalgae and several corals as part of a PhD thesis. Three new compounds have been isolated and described [34], and their bioactivity experimentally tested on corals results in severe coral bleaching.

### 5.6. Microalgae and Cyanobacteria

**Figure 20:**
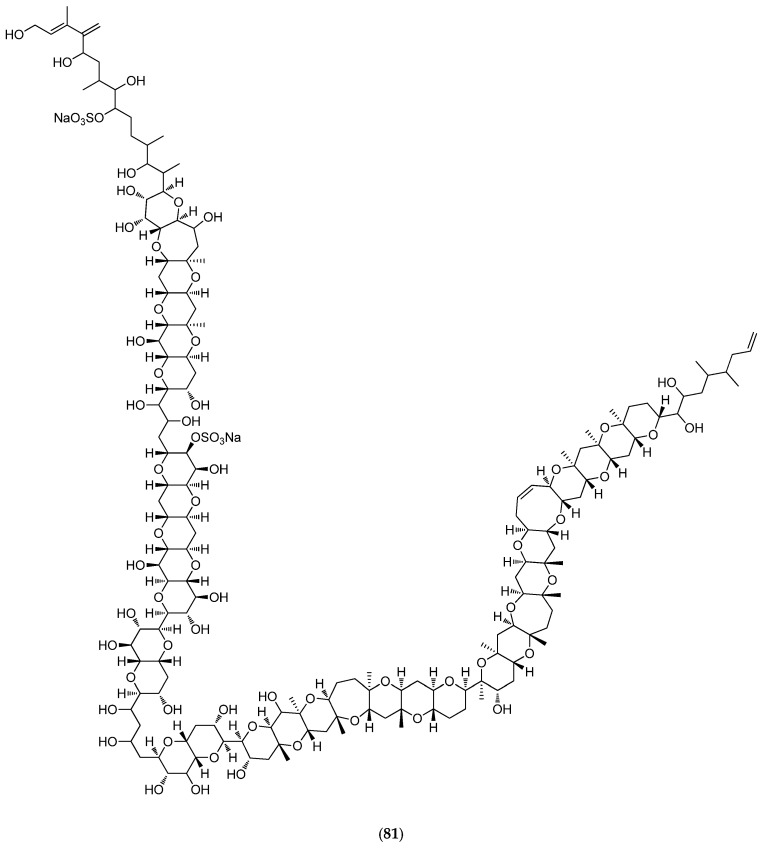
Structure of maitotoxin (**81**).

Less than 1% of described marine phytoplanktonic species are known to produce potent toxins [157]. Microalgal benthic dinoflagellates species are involved, including *Gambierdiscus toxicus* that produces maitotoxin (**81**, Figure 20). *In vivo* toxicity in mice indicates that intraperitoneal injection of 0.13 µg/kg is lethal [158]. A culture of ca. 4000 L of dinoflagellate cells is needed for the purification of 20 mg of MTX, enough to kill about four million mice. MTX activates both voltage-sensitive and receptor-operated calcium channels in the plasma membrane, thus inducing calpain protease that rapidly leads to cell death. MTX has since been considered as a useful tool for investigating the physiological and pathophysiological roles of calpain in neuronal cells [159].

**Figure 21:**
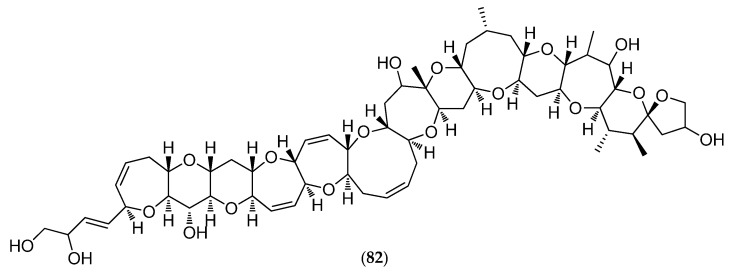
Structure of Pacific ciguatoxin-1 (P-CTX-1) (**82**).

Species belonging to the genus *Gambierdiscus* have also been shown to produce ciguatoxin analogues (CTX analogs) causing ciguatera fish poisoning (CFP). New Caledonia is a CFP-endemic area. CFP constitutes a global health problem with more than 50,000 people worldwide affected by this disease annually. CTXs arise from biotransformation/metabolization of CTX analogs through the food chain: from herbivorous fish that accumulate dinoflagellate toxins by eating dead coral and marine algae to carnivorous fish [157]. In Pacific areas, the principal and most potent CTX is Pacific ciguatoxin-1 (**82**, Figure 21). CTXs are potent activators of voltage-sensitive sodium channels and cause an increase in neuronal excitability and neurotransmitter release [160]. In New Caledonia, traditional remedies are commonly employed in the treatment of CFP: 90 plant species have been catalogued as useful CFP remedies, including the leaves of *Heliotropium foertherianum* used to prepare the most popular herbal remedy [161].

A study conducted by D. Laurent *et al.* following the observation of many cases of seafood poisoning on Lifou Island between 2001 and 2005 (Loyalty Islands, New Caledonia, Figure 1), indicates that bloom-forming cyanobacteria, such as the filamentous *Trichodesmium erythraeum*, can also produce CTX-like compounds [160].

In line with programs exploring the biodiversity of planktonic species such as OCEANOMICs (see [162] for details), the project AMICAL (Aquaculture of MIcroalgae in New CALedonia, [163]) instigated by IFREMER-New Caledonia, the Physiology and Biotechnology of Algae (PBA) laboratory (Nantes, mainland France) and ADECAL (Caledonian Economic Development Agency-Nouméa, [164]), aims to develop industrial microalgal production based on indigenous microalgae species isolated along the mainland coasts (Figure 1). Partners of this program will transfer the cultivation and extraction techniques to selected algal species for industrial programs in New Caledonia targeting the animal nutrition market and high added-value compounds (cosmetics, health food, *etc.*).

### 5.7. Other Biological Sources

Statistically “minor” phyla in terms of the number of studies on local species include prokaryotes, several invertebrate phyla, and snakes as the sole vertebrate representatives.

#### 5.7.1. Prokaryotes and Fungi

##### *Micrococcus* *Luteus*

**Figure 22:**
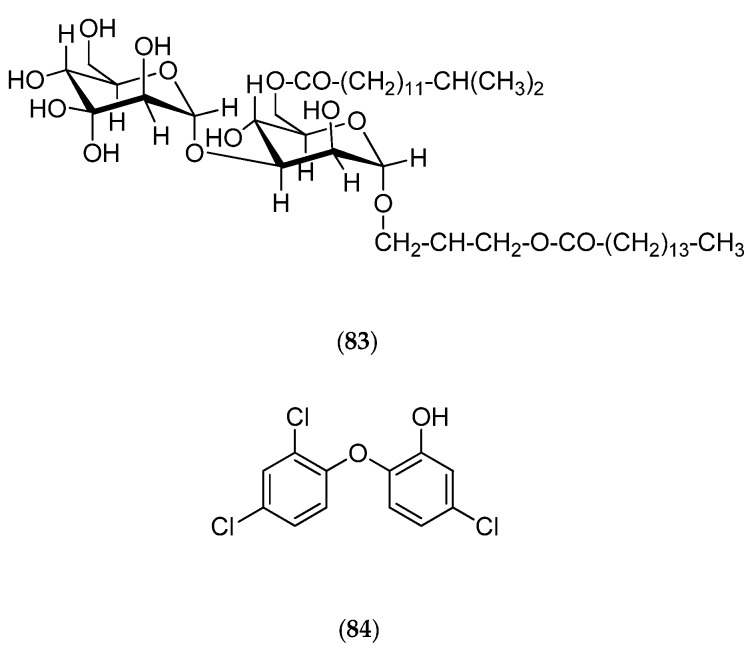
Structure of lutoside (**83**) and triclosan (**84**).

*Micrococcus luteus* is a Gram+ bacterium that was isolated from *Xestospongia exigua* sponges collected off Nouméa in the Southwest Pacific. Chemical studies reveal that this bacterium can produce lutoside (**83**, Figure 22), an acyl-1-(acyl-6′-mannobiosyl)-3-glycerol, and also triclosan (**84**, Figure 22) (2,4,4′-trichloro-2′-hydroxydiphenylether), with both molecules presenting antibacterial activities [165,166].

##### Bacteria from *Pseudoalteromonas* and *Vibrio* Genus

Along the west coast of New Caledonia, 205 environmental samples were collected on a variety of surfaces (sediments, intertidal rocks, invertebrates, plants, fish and biofilms found on organic substrates). This sampling led to the isolation of 493 marine bacteria [167,168]. Studies were at first performed to assess their ability to produce exopolysaccharides (EPSs). Among them, the new strain *Vibrio neocaledonicus* sp. nov. (NC470), isolated from a biofilm found on Holothuroidea in St. Vincent Bay, has demonstrated the production of EPSs that have a high N-acetyl-hexosamine and uronic acid content with a low amount of neutral sugars. Preliminary experiments conducted on these EPSs have shown high metal-binding capacity [167]. Further studies have shown that four other strains (NC282, NC412, NC272 and NC120), which belong to the genus *Pseudoalteromonas*, have antibacterial potential against reference and multidrug-resistant pathogen strains such as *Staphylococcus aureus*, *Pseudomonas aeruginosa*, *Escherichia coli* and *Enterococcus faecalis* [168].

##### *Acremonium* *Neocaledoniae*

**Figure 23:**
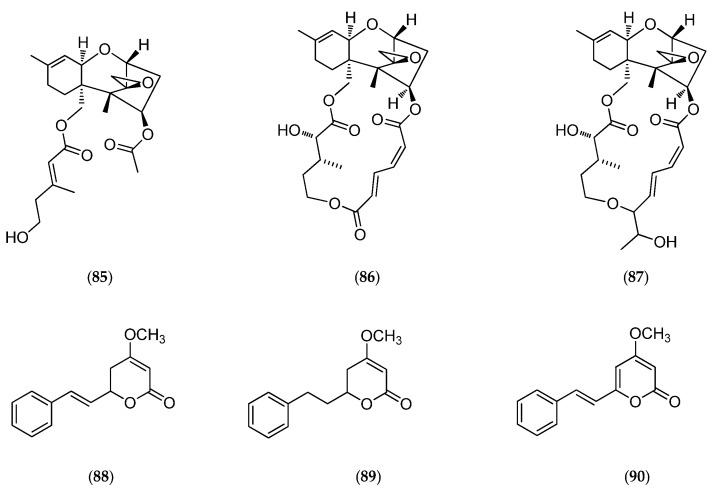
Structure of verrol 4-acetate (**85**), verrucarine A (**86**), isororidine A (**87**), kawain (**88**), 7,8-dihydrokawain (**89**) and 5,6-dehydrokawain (**90**).

The culture of the marine fungus *Acremonium neocaledoniae* (moniliaceae), collected on driftwood in the southwestern lagoon of New Caledonia, led to the production of verrol 4-acetate (**85**, Figure 23) [169], a new cytotoxic metabolite sesquiterpene trichothecene, along with known trichothecenic mycotoxins (e.g., verrucarine A (**86**, Figure 23), isororidine A (**87**, Figure 23) and some previously described styrylpyrones, such as kawain (**88**, Figure 23), 7,8-dihydrokawain (**89**, Figure 23) and 5,6-dehydrokawain (**90**, Figure 23) [170].

#### 5.7.2. Venomous Cone Snails

**Figure 24:**
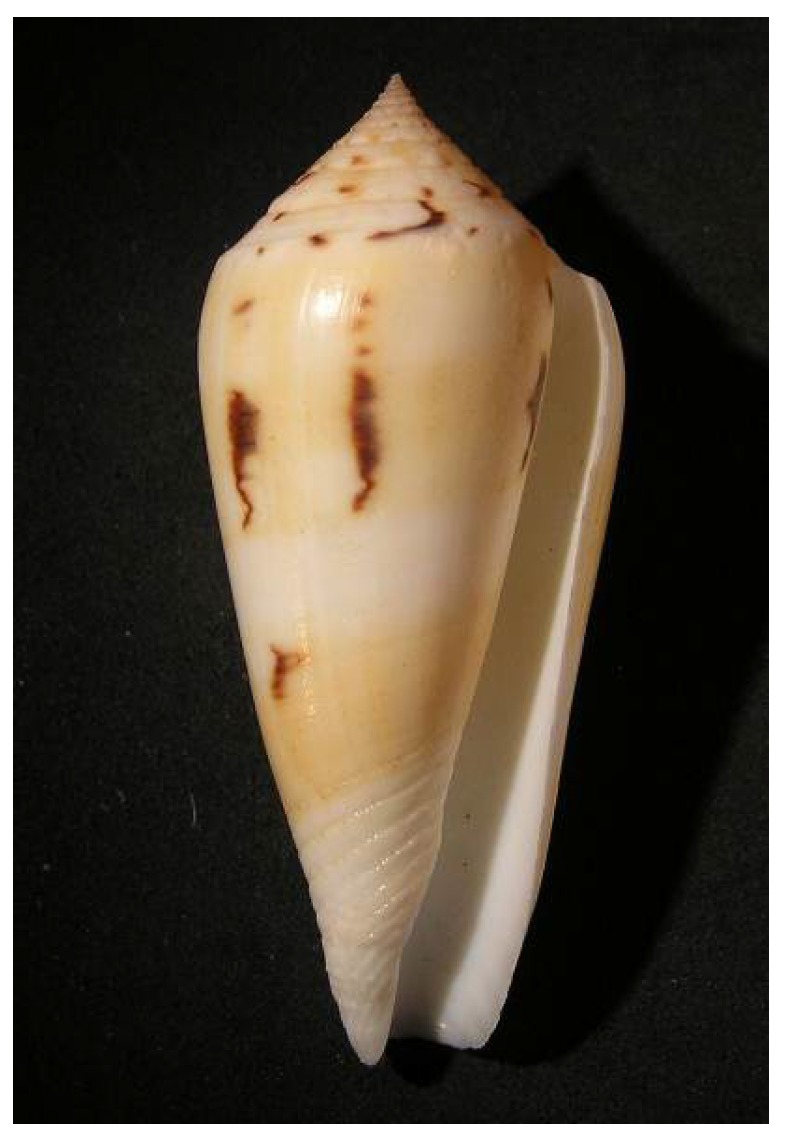
*Conus consors*. Photo Jan Delsing [175].

Venomous marine cone snails (Conoidea) are neogastropod mollusks that predate on marine worms and small benthic invertebrates (thick-shelled snails, e.g., *Conus textile*) or that actively “hunt” fish (thin-shelled snails, e.g., *Conus geographus*) primarily by inflicting cocktails of peptide neurotoxins produced by venom glands via their modified harpoon-like radula (see review in Kaas and Craik, 2014 [171]). Fish-hunting cone snails may switch from prey-stimulated to defensive envenomation strategies as shown by the use of different and more potent “high threat” neurotoxins if threatened, the latter being regarded as a specialization in response to predation pressure from fish and cephalopods [172]. In addition, *C. geographus* (common in New Caledonia) diffuses a “cloud” of fish-like insulin that literally "tetanizes" the fish by eliciting hypoglycemic shock prior to envenomation [173], a possible metabolic cost-saving feature. Pharmacological applications of cone snail venoms have stemmed from the work of Olivera’s group on *Conus magus*, which inspired the biosynthesis of the most potent pain-killer on the market (ziconotide, Prialt^®^, Dublin, Ireland), claimed to be 1000 times more potent than morphine for the sedation of terminally ill cancer patients. The CONCO cone snail genome project for health funded by the European Commission was launched in 2005 to study the toxins composing the venom of the cone snail *Conus consors* (Figure 24) from the Chesterfield Islands using genomic, transcriptomic and proteomic approaches. The mitochondrial genome of *C. consors* has now been sequenced and gene annotated. The authors report the presence of a novel 700 bp control region absent from the hitherto known mitochondrial genomes of cone shells [174]. The species richness of venomous cone mollusks in New Caledonia is exceptional and prompts further investigations in the molecular biology, pharmacology and ecology of this fascinating group.

### 5.8. Vertebrates: Venomous Marine Snakes

Globally, snake venom studies are of primordial importance for the design of novel antivenoms, for research tools in neurophysiology, and for a better understanding of the adaptive history of reptiles.

**Figure 25:**
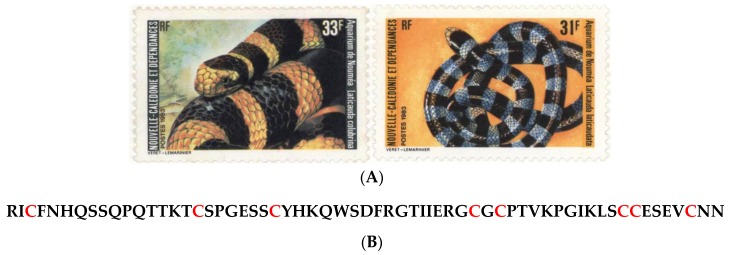
(**A**) *Laticauda colubrina* and *Laticauda laticaudata*. Postage stamps issued by the *Office des Postes et Télécommunications* (1983) (courtesy of a private collection); (**B**) Amino-acid sequence of erabutoxin b (**91**). Half cysteine residues are noted in red. One-letter codes for amino acids: C, cysteine; D, aspartate; E, glutamate; F, phenylalanine; G, glycine; H, histidine; I, isoleucine; K, lysine; L, leucine; N, asparagine; P, proline; Q, glutamine; R, arginine; S, serine; T, threonine; V, valine; W, tryptophan; Y, tyrosine. Adapted from [179].

Like Australia, New Caledonia has no terrestrial venomous snakes, and Gail and Rageau established the first census of venomous marine snakes from the Elapidae family in 1955 [176]. Of the marine snakes that have been investigated by toxicologists, the most common ones are *Laticauda laticaudata* and its congener *Laticauda colubrina*, both highly venomous snakes that essentially hunt small fish underwater at night and rest on land during the day (Figure 25A).

*L. laticaudata* and *L. colubrina* rank, respectively, 9th and 46th among the 163 most venomous snakes (terrestrial and marine species of all continents). Being related to well-known Asian cobras and African mambas (Elapidae), but being much easier to handle (the mouth is very small and the hooks are adapted to bite prey during the swallowing process), laticaudas are an excellent model for toxicologists. A major component, erabutoxin b (**91**, Figure 25B) from *Laticauda semifasciata* binds with high affinity to muscular nicotinic acetylcholine receptors (nAChRs), but with low affinity to neuronal alpha-7 nAChRs and inhibits acetylcholine from binding to the receptor, thereby impairing neuromuscular transmission [177]. As a result, it produces peripheral paralysis by blocking neuromuscular transmission at the postsynaptic site. The first cDNA studies undertaken as part of a collaboration between the *Centre d'Etudes Nucléaires* *de Saclay* and Tokyo University [178] later included New Caledonian snakes in a review of the evolution of Elapid snake venoms [179].

*Aipisurus laevis* is a fully pelagic species, collected in surface waters of the lagoon in the Southern Province. *A. laevis* ranks 10 out of the 163 known most “globally dangerous” snakes, and 30 out of 163 in terms of toxicity (LD_50_ by injection in mice). Two cDNAs of short-chain neurotoxins were cloned and sequenced by Ducancel *et al.*, (1990) [180], and compared with similar work on erabutoxin b (**91**, Figure 25B) from *Laticauda* snakes.

## 6. Recent Advances on Selected New Caledonian Marine Natural Products

Following their discovery in the course of ongoing New Caledonian scientific programs, and after promising results were revealed by preliminary biological screening and spectral characterization, further biological and chemical investigations on several molecules have been pursued to carry out (i) total synthesis; (ii) synthesis of chemical derivatives selected on the basis of structure-activity relationship (SAR) studies; (iii) determination of the mechanism of action; (iv) determination of additional bioactivities including *in vivo* and receptor studies. These studies and relevant updates are listed in Table 6.

Despite recent advances in developing therapeutic strategies, cancer is still one of the leading causes of death worldwide. Cancer is a group of diseases characterized by the uncontrolled growth and spread of abnormal cells. Drug-induced programmed cell death holds promise for cancer therapeutic approaches. Apoptosis is one of the well-known pathways used by cells to die. It is a morphological event characterized by chromosomal DNA fragmentation, nuclear disintegration, cell shrinkage, translocation of phosphatidyl serine moieties to the outer membrane leaflet, and membrane blebbing [209]. Numerous anticancer drugs, both on the market and in development, have apoptosis-modulating properties (e.g., Yondelis^®^, Madrid, Spain, has been recently approved by the American Food and Drug Administration (FDA) to treat patients with advanced soft tissue sarcoma [210]). Apoptogens are agents that can induce rapid death by modulating the apoptosis pathway. Numerous marine natural products reported in Table 6 are apoptogenic compounds associated with antitumor activity.

**Figure 26:**
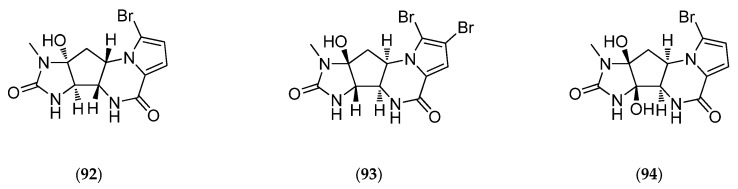

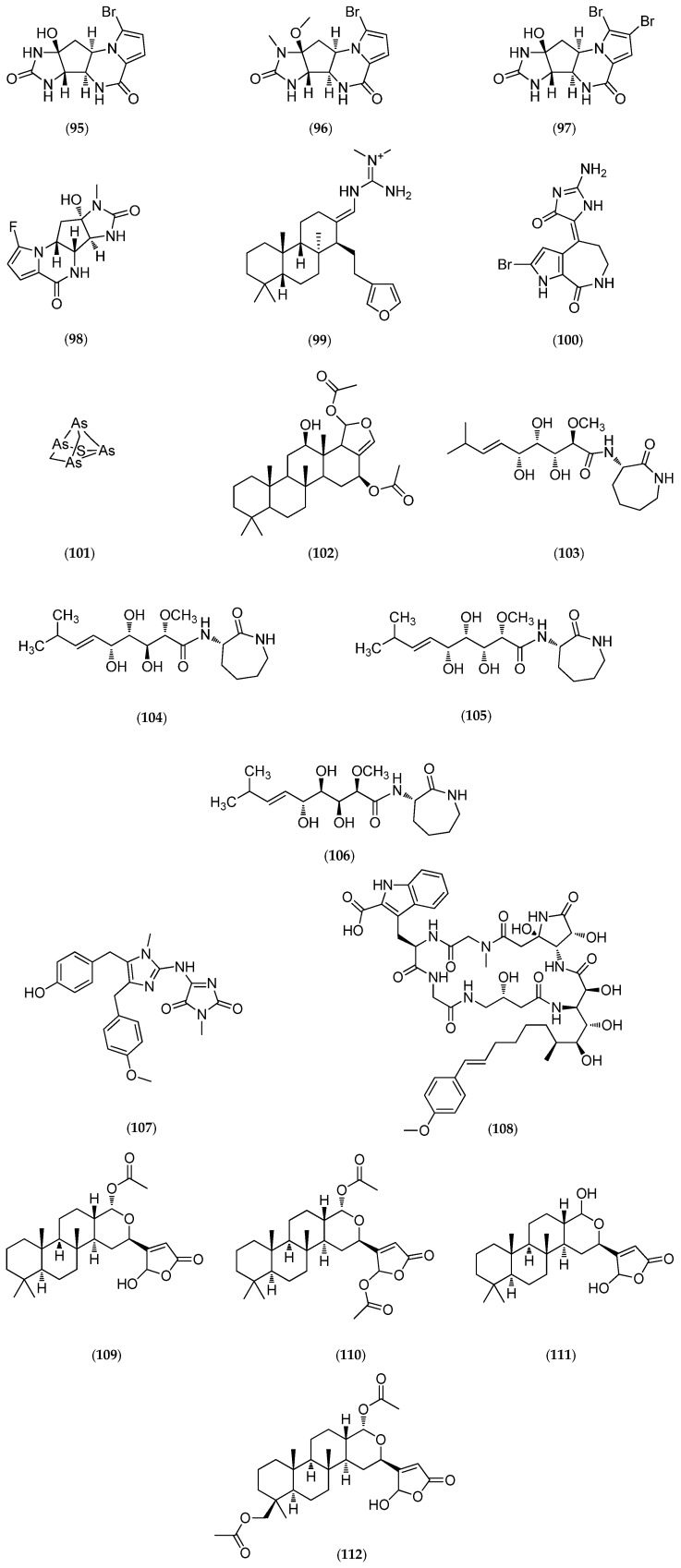

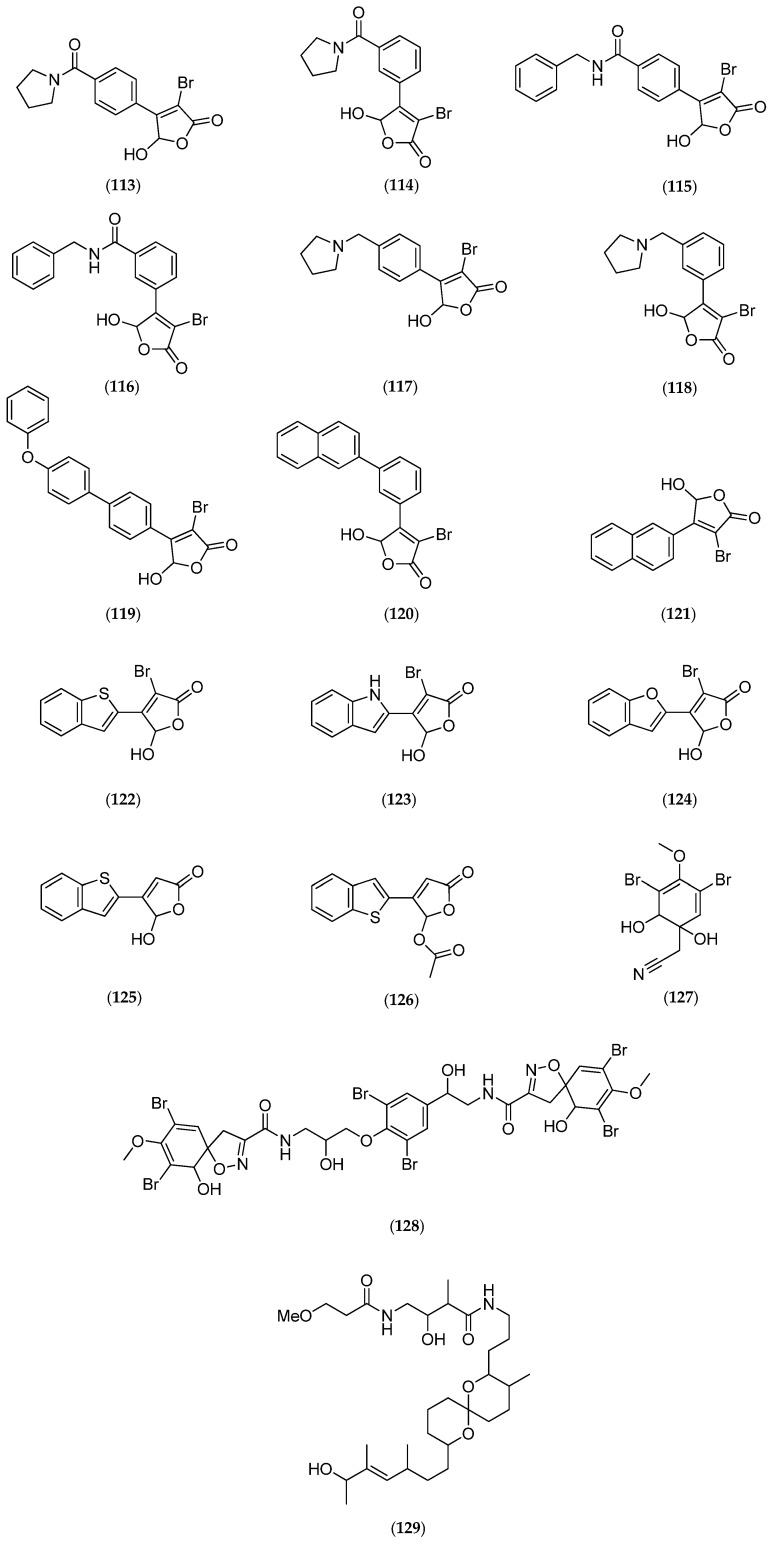

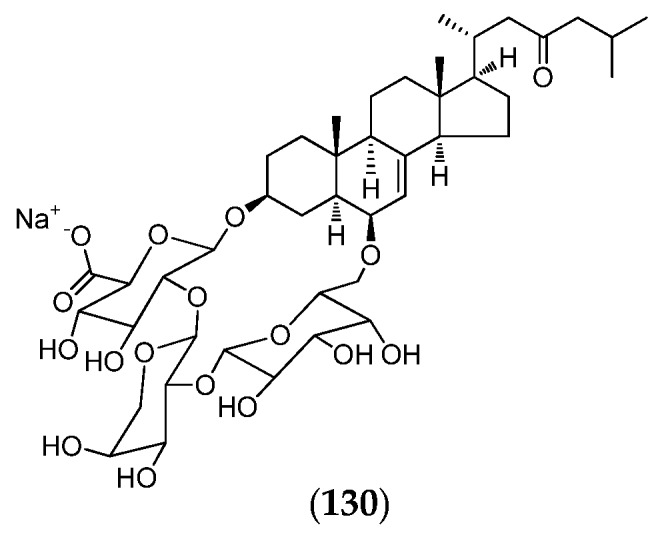
Structure of agelastatin A–F (**92**–**97**), 13-debromo-13-trifluoromethyl agelastatin A (**98**), suvanine (**99**), isohymenialdisine (**100**), monosulfide arsenicin A (**101**), heteronemin (**102**), bengamide E (**103**) and analogs: 2,3-bis-*epi*-bengamide E (**104**), 2-*epi*-bengamide E (**105**) and 3,4-bis-*epi*-bengamide E (**106**), naamidine A (**107**), microsclerodermin A (**108**), petrosaspongiolide M (**109**), natural analogs of petrosaspongiolide M (**110**–**112**), first generation of synthetic petrosaspongiolide M analogs (**113**–**120**), second generation of synthetic petrosaspongiolide M analogs (**121**–**126**), aeroplysinin-1 (**127**), fistularin-3 (**128**), analogs of bistramide A (**129**), adapted from [206] and luzonicoside A (**130**).

Agelastatins, members of the chemically diverse pyrrole-imidazole alkaloids (PIA), are among the best examples of this class of molecules. Since the discovery of agelastatin A (**92**, Figure 26) in 1993 by Pietra *et al.*, in the New Caledonian coral sea sponge *Agelas dendromorpha*, more than ten different research groups have reported innovative solutions to synthesize it. As reported in Han *et al.*, 2013, recent development of a concise, stereo-controlled, and biosynthetically inspired strategy to synthesize agelastatin alkaloids and a new synthetic methodology for azaheterocycle synthesis has given rise to many agelastatin derivatives. These developments also facilitate the first side-by-side testing of all known agelastatin alkaloids for their ability to induce cell death in various cancer cells [181]: U-937 (lymphoma), HeLa (cervical carcinoma), A549 (non-small-cell lung carcinoma), BT549 (breast carcinoma), and IMR90 (immortalized lung fibroblasts) human cell lines. Agelastatins A (**92**, Figure 26) and D (**95**, Figure 26) both induce dose-dependent apoptosis (in particular cell membrane permeabilization, activation of procaspase-3 to active caspase-3 and proteolytic cleavage of poly(ADP-ribose) polymerase (PARP)) and exhibit dose-dependent G2/M cell cycle arrest in synchronized U-937 cells without affecting tubulin dynamics within cells [181]. In addition, the potency of all agelastatins (**92**–**97**, Figure 26) has been evaluated in five human blood cancer cell lines. Interestingly, agelastatin C (**94**, Figure 26) is only weakly active and agelastatin F (**97**, Figure 26) is inactive against the tested cell lines. Agelastatin A (**92**, Figure 26) is remarkably active, particularly against CEM from acute lymphoblastic leukemia and against Daudi from Burkitt’s lymphoma (values reported in Table 6) and specifically target these white blood cell lines over normal red blood cells: it is 16,650 times more active on cancer cells than on normal cells! These results are in line with a previous study showing the inhibition of osteopontin-mediated neoplastic transformation (notably by β-catenin inhibition) and metastasis by agelastatin A (**92**, Figure 26) in MDA-MB-231 and MDA-MB-435 human breast cancer cell lines [211]. Therefore, agelastatins exhibit considerable potential as antitumor drugs that can simultaneously inhibit cancer cell growth as well as act as a potent anti-metastatic drug [211]. However, the mechanism through which agelastatin A (**92**, Figure 26) causes this cellular effect, particularly cycle arrest, still needs to be elucidated. Identification of potential intracellular targets of agelastatins can be performed by affinity chromatography on immobilized agelastatin. This approach has previously provided valuable insights into the cellular targets of various protein kinases inhibitors including hymenialdisine (**12**, Figure 5) [212].

Among the molecules reported in Table 6, heteronemin (**102**, Figure 26), a marine sesterterpene, has been shown to induce apoptosis in A-498 human renal carcinoma cells by downregulating apoptosis inhibitors Bcl-2 and Bcl-xL and upregulating the death agonist Bax, leading to the disruption of the mitochondrial membrane potential and the release of cytochrome c from mitochondria [192]. As also reported for agelastatins, these effects are associated with the activation of caspases (3/8 and 9), followed by PARP cleavage. Interestingly, the same study showed that heteronemin (**102**, Figure 26) also induces autophagy in A-498 cells, but the inhibition of autophagy enhances the anticancer effect of heteronemin (**102**, Figure 26) in A-498 cells. This result suggests that the combination of heteronemin (**102**, Figure 26) with autophagy inhibitors further enhances its therapeutic effects for cancer treatment [192]. Therefore, marine molecules can represent chemical tools for the exploration of novel combined therapeutic strategies. Petrosaspongiolide M (**109**, Figure 26) may also represent an equally valuable chemical tool for its capacity to modulate intracellular proteolysis through dual inhibition of the immunoproteasome and autophagy [202].

Synergistic effects (e.g., treatment with heteronemin (**102**, Figure 26) and inhibitors of autophagy such as chloroquine) of marine products and therapeutic drugs on disease-related phenotypes highlight the concept of combination therapy. The cytotoxicity of new marine molecules should be tested alone or in combination with known anticancer compounds to overcome drug resistance, as reported in Elmallah and Micheau (2015) for the resistance of cancer cells to TNF-related apoptosis inducing ligand (TRAIL)-induced cell death [209]. TRAIL is a molecule that selectively kills—via apoptosis—transformed and cancer cells, but not most normal cells [213]. Manzamine A, an alkaloid originally isolated from an Okinawan sponge *Haliclona* sp., may restore TRAIL-induced apoptotic cell death in the TRAIL-resistance pancreatic AsPC-1 cell line via inhibition of glycogen synthase kinase-3β (GSK-3β) and subsequent inhibition of the survival factor NF-κB [209]. It will thus be very interesting to evaluate the TRAIL-sensitizing activity of agelastatin A (**92**, Figure 26); which is known to inhibit GSK-3β with an IC_50_ of 12 µM [44] (manzamine A has an IC_50_ for GSK-3β inhibition of 10 µM [214]). All new marine natural products, including those from biodiversity hotspots such as the New Caledonia archipelago, represent putative new hope for cancer treatments, in particular for overcoming defects in apoptosis signaling.

As highlighted above, targeting apoptotic pathways may have a direct role in inducing tumor cell death, as confirmed by recent advances on marine molecules found in New Caledonia. In addition to agelastatins and heteronemin (**102**, Figure 26), naamidine A (**107**, Figure 26), microsclerodermin A (**108**, Figure 26), aeroplysinin-1 (**127**, Figure 26), fistularin-3 (**128**, Figure 26) have been shown to induce apoptosis (Table 6). Cancer is the principal target of the marine molecules released on the market today. Of the seven marine compounds currently on the market, only three (Prialt^®^, Dublin, Ireland; Yondelis^®^, Madrid, Spain and Lovaza^®^, London, UK) were not chemically modified to become drugs [215,216]. The four other molecules underwent lead optimization during the various different stages of their development. Developments in structural chemistry must be followed by the characterization of biological targets to optimize therapeutic applications.

## 7. Conclusions

Though far from being exhaustive, this review outlines 40 years of exciting research on the chemodiversity of marine organisms, ranging from microbes and invertebrates to vertebrates, from microalgae to macroalgae and halophytes, belonging to very different biota in association with the complex coral reef systems of New Caledonia.

Traditionally “interesting” lead groups such as sponges, cnidarians and ascidians have been intensely investigated because they not only provide the most interesting array of original chemical structures, but they also show the most potent anticancer, anti-inflammatory and antibiotic properties. Other groups have occasionally led to original and stimulating research: echinoderms, mollusks, *etc.* This review focused on novel products although several studies have been performed on New Caledonian marine species leading to the rediscovery of some secondary metabolites (e.g., *Acremonium neocaledoniae*, p. 41).

The fact that “minor” groups have been largely left aside does not reflect their lack of intrinsic interest for chemical exploration, but rather the technical difficulties to collect or cultivate them in New Caledonia. Only about 2% of marine bacteria can be cultivated using classical growth media. Some small invertebrates require time-consuming field and laboratory work to get enough molecular material to work on (e.g., bryozoans), are haphazardly encountered (e.g., many “naked” nudibranch and opistobranch mollusks) or are on an endangered species list, *etc.* Noteworthy are independent investigations on different models: e.g., biomaterials from scleractinian corals for maxillofacial replacement of bone tissue [217] or from oyster nacre as an osteoinductor in odontology [218]. Another example is that of the endemic *Nautilus macromphallus* cephalopod excretory metabolism. This metabolism relies on a unique and seemingly very stable symbiosis with specific bacterial symbionts to produce molecular nitrogen in the chambered shell and regulate the buoyancy [219].

Figure 2 indicates that the “golden age” of traditional screening for novel molecules and bioactivity has ceased after several periods of intense investigation. This reflects the successive explorations of new “territories”, e.g., sea mounts, outer reef slopes and deep benthos in general. Based on what has been achieved thus far, taking into account the exceptional richness of the local biota and the growing need to develop local economy in a balanced and sustainable way, it appears necessary to develop a permanent, reliable and collaborative research and development pipeline linking field exploration to:

-drug development, including (i) isolation of active principles; (ii) high-throughput bioactivity screening; (iii) structure-activity investigations and structural elucidation; (iv) cultivation technologies or bioinspired synthesis; (v) clinical trials and beyond;
-aquaculture focusing on the treatment of locally grown species of prawns and oyster varieties that are sensitive to seasonal blooms of toxigenic bacteria and microalgae.

Molecular approaches have now come of age, making new biological models more attractive and promising. New Caledonia represents a living laboratory with its unique source of marine organisms that have not been investigated to date. There is now a need to better understand the relationships between host organisms and their microbial flora. This requires on-site operations and specialized equipment (aquaria, cultivation facilities for microorganisms). 

Finally, in the general context of “blue growth”, New Caledonia is ideally located for investigating the production of third-generation biofuels and of high value-added products (e.g., cosmetics, nutraceuticals) via the biomass of microalgal primary producers.

New Caledonia is a treasure chest for scientists to explore, but is also fragile. With the perspective of climate change due to global warming and emerging anthropogenic forcings, the sustainability of the sources of molecules, including resident bacteria, must take precedence. New Caledonian coral reefs—of which some portions were added to UNESCO’s World Heritage list in 2008—must be protected for the livelihood of resident populations, as well as for biological inspiration for the sciences and the arts. Extensive studies of their chemodiversity should contribute to their protection.

## Figures and Tables

**Table 1:** Field observations and crude biological tests for preliminary screening.

Biological Model	Species	Type of Activity Tested	Reference
Bacteria	*Escherichia coli*, *Pseudomonas aeruginosa*, *Staphylococcus aureus*, *Streptococcus faecalis (*now *Enterococcus faecalis)*, *Vibrio anguillarum*	Antibacterial	[26]
Fungi	*Candida albicans*, *Candida tropicalis,* *Helminthosporium graminearum*, *Helminthosporium turcicum*, *Penicillium italicum*, *Phytophthora parasitica*, *Pyricularia oryzae*	Antifungal	[26]
Brine shrimp larvae	*Artemia salina*	Cytotoxicity	[27]
Fish	*Gambusia affinis*	Neuro/Cytotoxicity	[28,29]
Urchin eggs	*Echinometra mathaei*	Cytotoxicity	[30]
Insect	*Hypothenemus hampei*	Insecticide	[31]
Mite	*Rhipicephalus microplus (*formerly*, Boophilus microplus)*	Acaricide	[32]
Algae	*Ceramium codii*	Anti-fouling	[33]
Coral	Algae	Allelopathy	[34]
Plant	*Amaranthus caudatus*	Anti-germinating	[35]

**Table 2:** Natural products isolated from New Caledonian sponges and their bioactivity.

Natural Product	Chemical Class	Biological Activity		Species	Reference
agelastatin A	unusual C11 alkaloid	antiproliferative kinase inhibitor	IC_50_ (µg/mL) KB 0.075, L1210 0.033 GSK-3β selective inhibitor (IC_50_ 12 µM).	*Agelas dendromorpha*	[41,42,43,44]
ageliferin	dimeric C11 oroidin-related alkaloid	neurotransmitter inhibitor	Active on SRIF 2.21 µM (K_i_ 2.47 µM); VIP 19.8 µM (K_i_ 63.8 µM).	*Agelas novaecaledoniae*	[45]
sceptrin			Active on SRIF 0.27 µM (K_i_ 0.30 µM); VIP 19.2 µM (K_i_ 61.8 µM).		
callipeltin A	cyclic depsidecapeptide	antiproliferative	IC_50_ (µg/mL) P388 < 3.3, NSCLC-N6 < 1.1, NSCLC-N6 C15 > 30, NSCLC-N6 C92 < 3.3, NSCLC-N6 C98 < 3.3, E39 < 1.1, M96 < 3.3	*Callipelta* sp.	[46,47]
		antiviral	Day 6 post-infection: CD_50_ 0.29 µg/mL and ED_50_ HIV-1 0.01 µg/mL (SI 29), AZT reference CD_50_ 50 µM and ED_50_ 30 nM.		
		antifungal	*Ca* 30 mm/100 µg/disk.		
callipeltin B		antiproliferative antiviral	IC_50_ (µg/mL) P388 < 3.3, NSCLC-N6 1.3, NSCLC-N6 C15 22.5, NSCLC-N6 C92 > 30, NSCLC-N6 C98 < 3.3, E39 > 10, M96 < 3.3 Inactive against HIV-1.		[47]
callipeltin C	acyclic depsidecapeptide	antiproliferative antifungal antiviral	IC_50_ (µg/mL) NSCLC-N6 53.5, E39 36.1 *Ca* 9 mm/100 µg/disk. Inactive against HIV-1.		[47]
callipeltoside A	glycoside macrolide	antiproliferative mechanism of action	IC_50_ (µg/mL) P388 15.26, NSCLC-N6 11.26 G1 cell cycle arrest in NSCLC-N6 cells.		[48]
callipeltoside B		antiproliferative	IC_50_ NSCLC-N6 15.1 µg/mL.		[49]
callipeltoside C			IC_50_ NSCLC-N6 30.0 µg/mL.		
corallistin A	porphyrin	antiproliferative	IC_50_ KB 10 µg/mL. Inactive *in vivo* and in tubuline assay.	*Corallistes* sp.	[50,51]
coscinosulfate	sulfated sesquiterpene	antiproliferative antimitotic antibacterial	Against Jurkat and HBL100 cells (data not shown). CDC25A inhibitor: IC_50_ 3 µM. *Sa* 12 mm/50 µg/disk.	*Coscinoderma mathewsi*	[52]
dibromocantharelline	C_11_ alkaloid	kinase inhibitor	IC_50_ GSK3-β 3 µM.	*Cymbastela cantharella (formerly Pseudaxinyssa cantharella)*	[44,53]
girolline	degraded C_11_ alkaloid	antiproliferative antimalarial	IC_50_ (µM) P388 0.06, P388/Dox 0.08, KB 0.21, T24 0.19 IC_50_ *Pf* 77-215 nM.		[54,55,56,57], [58,59,60,61,62]
pyraxinine	3-pyridylguanidine	anti-inflammatory	Inhibition of macrophagic NO synthase at 100 µM.		[63]
hymenialdisine	tricyclic pyrrole alkaloid	kinase inhibitor	IC_50_ (nM) CDK1/cyclin B 22, CDK2/cyclin A 70, CDK2/cyclin E 40, CDK5/p25 28, GSK-3β 10, CK1 35 and PLK-1 10,000.		[44,64]
methyl diacarnoate A	*epi*-dioxy norditerpene	antiproliferative	Inactive against KB cells.	*Diacarnus levii*	[65]
methyl 3-epinuapapuanoate		antimalarial antiproliferative	IC_50_ 7.4 and 7.2 µM on CQ-sensitive and CQ-resistant *Pf*, resp. *IC_50_* KB >> 20 µg/mL.		[65,66]
2-epimukubilin benzyl ester	*epi*-dioxy norsesterterpene	antiproliferative	IC_50_ KB 1.0 µg/mL.		[65]
methyl prenyldiacarnoate A			IC_50_ KB 3.3 µg/mL.		
methyl 2-epiprenyldiacarnoate A			IC_50_ KB 0.9 µg/mL.		
nortopsentin D	bis-indole alkaloid	antiproliferative	EC_50_ KB 0.014 µg/mL (permethylated derivative).	*Dragmacidon* sp.	[67]
arsenicin A	polyarsenic	antibacterial antifungal	10 µg/disk, *Sa*/*Ec*/*Ca*: 24/28/26 (mm) for arsenicin A, and 22/30/22 (mm) for gentamicin.	*Echinochalina bargibanti*	[68]
euryspongiol A1	polyhydroxylated 9,11-secosterol	antihistaminic	Reduction of histamine release by 26% (control 35%).	*Euryspongia* sp.	[69]
euryspongiol A2			Reduction of histamine release by 15% (control 35%).		
homophymine A	cyclodepsipeptide	antiviral antiproliferative	IC_50_ HIV-1 75 nM. IC_50_ (nM) KB 7.3, MCF-7 23.6, MCF-7R 22.9, HCT116 6.0, HCT15 22.5, HT29 70.0, OVCAR8 5.4, OV3 7.5, PC3 4.2, Vero 5.0, MRC5 11.0, HL60 24.1, HL60R 22.4, K562 24.0, PaCa 31.4, SF268 9.9, A549 8.3, MDA231 10.9, MDA435 39.0, HepG2 68.6, EPC 5.0	*Homophymia* sp.	[70,71]
homophymine B		antiproliferative mechanism of action	IC_50_ (nM) KB 18.0, MCF-7 16.8, MCF-7R 26.3, HCT116 13.8, HCT15 22.9, HT29 101.9, OVCAR8 8.0, OV3 9.9, PC3 6.2, Vero 8.6, MRC5 17.1, HL60 43.1, HL60R 36.7, K562 26.7, PaCa 62.0, SF268 17.2, A549 19.8, MDA231 17.0, MDA435 40.1, HepG2 99.0, EPC 8.0. Caspase-independent cell death pathway (HL60).		[71]
homophymine C		antiproliferative	IC_50_ (nM) KB 8.5, MCF-7 8.8, MCF-7R 10.8, HCT116 4.9, HCT15 19.2, HT29 62.8, OVCAR8 4.3, OV3 3.7, PC3 3.0, Vero 4.2, MRC5 16.8, HL60 23.0, HL60R 23.5, K562 22.5, PaCa 25.9, SF268 13.6, A549 8.3, MDA231 16.2, MDA435 35.0, HepG2 72.1, EPC 9.3		
homophymine D			IC_50_ (nM) KB 12.7, MCF-7 19.6, MCF-7R 37.7, HCT116 19.8, HCT15 43.2, HT29 81.3, OVCAR8 8.1, OV3 10.6, PC3 6.3, Vero 10.9, MRC5 16.9, HL60 29.6, HL60R 24.9, K562 35.3, PaCa 37.4, SF268 17.9, A549 13.8, MDA231 18.9, MDA435 49.9, HepG2 78.7, EPC 11.1		
homophymine E			IC_50_ (nM) KB 6.0, MCF-7 14.2, MCF-7R 15.6, HCT116 5.5, HCT15 27.2, HT29 35.1, OVCAR8 4.6, OV3 4.2, PC3 3.9, Vero 7.0, MRC5 9.5, HL60 23.3, HL60R 21.4, K562 22.2, PaCa 18.1, SF268 8.1, A549 9.6, MDA231 13.3, MDA435 38.3, HepG2 60.5, EPC 9.5		
homophymine A1			IC_50_ (nM) KB 7.1, MCF-7 12.4, MCF-7R 13.5, HCT116 6.1, HCT15 13.5, HT29 30.9, OVCAR8 5.1, OV3 5.5, PC3 3.7, Vero 6.1, MRC5 7.8, HL60 17.3, HL60R 11.1, K562 12.8, PaCa 19.2, SF268 6.3, A549 6.0, MDA231 8.4, MDA435 27.0, HepG2 91.4, EPC 7.8		
homophymine B1	cyclodepsipeptide	antiproliferative	IC_50_ (nM) KB 16.4, MCF-7 14.2, MCF-7R 12.3, HCT116 11.4, HCT15 14.1, HT29 93.8, OVCAR8 6.5, OV3 8.0, PC3 4.7, Vero 6.1, MRC5 10.2, HL60 18.7, HL60R 25.8, K562 16.6, PaCa 22.2, SF268 11.7, A549 8.6, MDA231 18.2, MDA435 29.5, HepG2 100.3, EPC 6.6	*Homophymia* sp.	[71]
homophymine C1		antiproliferative mechanism of action	IC_50_ (nM) KB 6.8, MCF-7 6.3, MCF-7R 5.4, HCT116 2.7, HCT15 17.2, HT29 38.2, OVCAR8 2.6, OV3 2.4, PC3 2.6, Vero 3.1, MRC5 8.0, HL60 14.6, HL60R 17.1, K562 11.9, PaCa 14.4, SF268 7.1, A549 6.2, MDA231 15.8, MDA435 20.3, HepG2 58.6, EPC 12.2 Caspase-independent cell death pathway (HL60).		
homophymine D1		antiproliferative mechanism of action	IC_50_ (nM) KB 10.6, MCF-7 3.5, MCF-7R 3.5, HCT116 1.8, HCT15 11.4, HT29 32.2, OVCAR8 1.6, OV3 1.4, PC3 1.4, Vero 1.8, MRC5 10.5, HL60 13.1, HL60R 21.9, K562 12.9, PaCa 17.6, SF268 7.9, A549 5.0, MDA231 11.1, MDA435 23.4, HepG2 80.4, EPC 7.7 Caspase-independent cell death pathway (HL60).		
homophymine E1		antiproliferative	IC_50_ (nM) KB 12.5, MCF-7 3.9, MCF-7R 7.1, HCT116 2.3, HCT15 10.1, HT29 31.8, OVCAR8 4.0, OV3 2.7, PC3 3.5, Vero 4.4, MRC5 12.3, HL60 20.5, HL60R 23.2, K562 17.8, PaCa 10.6, SF268 10.1, A549 11.4, MDA231 20.0, MDA435 37.0, HepG2 62.8, EPC 29.0		
12-*epi*-heteronemin	scalarane sesterterpene	farnesyltransferase inhibitor	Inactive on farnesyl transferase.	*Hyrtios erecta*	[72,73]
heteronemin			IC_50_ 3 µM.	*Hyrtios reticulata*	[73]
thorectolide	terpene	antiproliferative antiviral	IC_50_ KB 5.3 µg/mL. HIV-1 nucleocapside and integrase inhibitor 10 and 20 µg/mL, resp.	*Hyrtios* sp.	[74]
thorectolide monoacetate		antiproliferative anti-inflammatory	IC_50_ KB 0.3 µg/mL. Cobra venom PLA_2_ inhibitor 2 µM, bee venom PLA_2_ inactive.		
puupehenone	merosesquiterpene	antiproliferative antifungal antibacterial antimalarial	IC_50_ KB 0.8 µg/mL. *Ct* 12 mm/50 µg/disk. *Sa* 12 mm/50 µg/disk. IC_50_ (µg/mL) *Pf* F32 0.6, FcB1 2.1 and PFB 1.5		[75,76]
dipuupehedione		antiproliferative antifungal antibacterial	IC_50_ KB 3 µg/mL. Inactive against *Ct.* Inactive against *Sa.*		[75]
15α-methoxypuupehenol		antiproliferative antibacterial antifungal antimalarial	IC_50_ KB 6 µg/mL. *Sa* 7 mm/1 µg/disk. *Ct* 9 mm/50 µg/disk. IC_50_ (µg/mL) *Pf* F32 0.4, FcB1 1.4 and PFB 1.2		[76]
pentaprenylhydro-quinone 4-sulfate	terpene	NPY inhibitor kinase inhibitor antiviral	IC_50_ 50.8 µg/mL. IC_50_ TPK 8 µg/mL. HIV-1 integrase: 65% inhibition at 1 µg/mL.	*Ircinia* sp.	[77]
hexaprenylhydro-quinone 4-sulfate		kinase inhibitor	IC_50_ TPK 4.0 µg/mL.		
heptaprenylhydro-quinone 4-sulfate			IC_50_ TPK 8.0 µg/mL.		
leucascandrolide A	macrolide	antiproliferative antifungal	IC_50_ (µg/mL) KB 0.05, P388 0.25 *Ca* 26 mm/40 µg/disk.	*Leucascandra caveolata*	[78]
leucascandrolide B			IC_50_ (µg/mL) KB 5, P388 >10 Inactive against *Ca.*		[79]
microsclerodermin A	cyclic hexapeptide	antifungal	*Ca* 2.5 µg/disk.	*Microscleroderma* sp.	[80]
microsclerodermin B					
sphinxolide	macrolide	antiproliferative antifungal	IC_50_ (ng/mL) NSCLC-N6 27, P388/Dox 0.33, P388 4.1, KB 7.0, HT29 115 IC_90_ values < 2 ppm against *Pc*, *Pci*, *Pr*, *Pv*, *Bc*, *Po*, *Fr*, *Aa*, *Rs*, *Ph*, *Sn*, *Hg* and *Un.*	*Neosiphonia superstes*	[81,82]
sphinxolide B			IC_50_ (ng/mL) KB 0.03, P388 3.1, P388/Dox 0.02, NSCLC-N6 16, HT29 2.4 IC_90_ values < 2 ppm against *Pc*, *Pci*, *Pr*, *Pv*, *Bc*, *Po*, *Fr*, *Aa*, *Rs*, *Ph*, *Sn*, *Hg* and *Un.*		
sphinxolide C			IC_50_ (ng/mL) KB 40, P388 40, P388/Dox 30, NSCLC-N6 30, HT29 30 IC_90_ values < 2 ppm against *Pc*, *Pci*, *Pr*, *Pv*, *Bc*, *Po*, *Fr*, *Aa*, *Rs*, *Ph*, *Sn*, *Hg* and *Un.*		
sphinxolide D		antiproliferative	IC_50_ (ng/mL) NSCLC-N6 60, P388/Dox 8, P388 3, KB 3.0, HT29 22		[81]
sphinxolide E			NCI screening: 60 human cell lines (9 cancer types: leukemia, lung, colon, brain, melanoma, ovarian, renal, prostate and breast). Sphinxolides F and G: less potent by 10-100 times compared to E. Same degree of cell line selectivity.		[83]
sphinxolide F					
sphinxolide G					
superstolide A			IC_50_ (µg/mL) KB 0.02, P388 0.003, P388/Dox 0.02, NSCLC-N6-L16 0.04, HT29 0.04		[84]
superstolide B			IC_50_ (µg/mL) KB 0.005, P388 0.003, NSCLC-N6-L16 0.039		[85]
neosiphoniamolide A	cyclic depsipeptide	antifungal	IC_90_ *Po* and *Hg* 5 ppm, > 5 ppm against *Pc*, *Pci*, *Pr*, *Pv*, *Bc*, *Fr*, *Aa*, *Rs*, *Ph*, *Sn* and *Un.*		[82]
nepheliosyne A	C_47_ polyoxygenated acetylenic acid	antiproliferative	IC_50_ (µM) K562 200, U266 170, SKM1 250, Kasumi 200	*Niphates* sp.	[86]
nepheliosyne B			IC_50_ (µM) K562 150, U266 200, SKM1 > 250, Kasumi 150		
gelliusine A	racemic tris-indole alkaloid	antiproliferative	10 < IC_50_ (µg/mL) < 20 against KB, P388, P388/Dox, HT29 and NSCLC-N6 cells.	*Orina* sp. (formerly *Gellius* sp.)	[87,88]
		serotoninergic activity	Serotonin agonist (10-100 µM). SRIF (100% displacement) and NPY (90%) active at 5 µg/mL. Bradykinin receptor 100%. Inactive on NK3, AMPA, CGRP, galanin, glycine, NT and VIP-binding assays.		
gelliusine B	racemic tris-indole alkaloid	serotoninergic activity	Bradykinin receptor 93% and NPY 62%. Inactive on NK3, AMPA, CGRP, galanin, glycine, NT and VIP-binding assays.	*Orina* sp. (formerly *Gellius* sp.)	[87,88]
gelliusine E	racemic bis-indole alkaloid		Inactive on serotonin receptor. SRIF (87% displacement) active at 5 µg/mL. NPY 63% and bradykinin receptor 63%. Inactive on NK3, AMPA, CGRP, galanin, glycine, NT and VIP-binding assays.		[88]
gelliusine F			Inactive on serotonin receptor. SRIF (91% displacement) active at 5 µg/mL. Bradykinin receptor 89% and NPY 67%. Inactive on NK3, AMPA, CGRP, galanin, glycine, NT and VIP-binding assays.		
petrosaspongiolide A	cheilantane-type sesterterpene	antiproliferative	IC_50_ NSCLC-N6 13.0 µg/mL.	*Petrosaspongia nigra*	[89]
petrosaspongiolide B			IC_50_ NSCLC-N6 14.8 µg/mL.		
petrosaspongiolide C			IC_50_ NSCLC-N6 0.5 µg/mL.		
petrosaspongiolide D			IC_50_ NSCLC-N6 5.2 µg/mL.		
petrosaspongiolide E			IC_50_ NSCLC-N6 4.5 µg/mL.		
petrosaspongiolide F			IC_50_ NSCLC-N6 8.7 µg/mL.		
petrosaspongiolide G			Inactive against NSCLC-N6.		
petrosaspongiolide H			IC_50_ NSCLC-N6 8.1 µg/mL.		
petrosaspongiolide I			IC_50_ NSCLC-N6 6.8 µg/mL.		
petrosaspongiolide J			IC_50_ NSCLC-N6 6.3 µg/mL.		
petrosaspongiolide K			IC_50_ NSCLC-N6 1.3 µg/mL.		
petrosaspongiolide L			IC_50_ NSCLC-N6 5.7 µg/mL.		
petrosaspongiolide M		anti-inflammatory	PLA_2_ inhibitors (10 µM) 71% bee venom, 11.5% *N. naja* venom, 12.3% porcine pancreas and 68.6% human synovial.		[90]
petrosaspongiolide N			PLA_2_ inhibitors (10 µM) 43.9% bee venom, 6.8% *N. naja* venom, 11.6% porcine pancreas and 44.0% human synovial.		
petrosaspongiolide P			PLA_2_ inhibitors (10 µM) 37.9% bee venom, 3.0% *N. naja* venom, 0.0% porcine pancreas and 60.9% human synovial.		
petrosaspongiolide Q			PLA_2_ inhibitors (10 µM) 12.5% bee venom, 4.2% *N. naja* venom, 0.0% porcine pancreas and 30.1% human synovial.		
petrosaspongiolide R			PLA_2_ inhibitors (10 µM) 18.8% bee venom, 1.0% *N. naja* venom, 0.8% porcine pancreas and 7.1% human synovial.		
phloeodictine A	guanidine alkaloid	antiproliferative antibacterial	IC_50_ KB 1.5 µg/mL. MIC (µg/mL) *Sf* 5, *Sa* 1, *Ec* 1, *Pa* 10	*Phloeodictyon* sp.	[91]
phloeodictine B			IC_50_ KB 11.2 µg/mL. MIC (µg/mL) *Sf* > 15, *Sa* 3, *Ec* 30, *Pa* > 30		
phloeodictine A1			IC_50_ KB 2.2 µg/mL. 2.6:1 mixture of phloeodictine A1 and A2. MIC (µg/mL) *Sa* 3, *Ec* 3, *Pa* 30, *Cp* 30, *Bf* 10 and *Pas* 10		[92]
phloeodictine A2					
phloeodictine A3			IC_50_ KB 3.5 µg/mL. 2.6:0.7:0.3 mixture of phloeodictine A3, A4 and A5. MIC (µg/mL) *Sa* 30, *Ec* 30, *Pa* > 30, *Cp >* 30, *Bf* > 30 and *Pas* > 30		
phloeodictine A4					
phloeodictine A5					
phloeodictine A6			IC_50_ KB 0.6 µg/mL. 1:1.4 mixture of phloeodictine A6 and A7. MIC (µg/mL) *Sa* 1, *Ec* 3, *Pa* 30, *Cp* 1, *Bf* 3 and *Pas* 3		
phloeodictine A7					
phloeodictine C1			IC_50_ KB 1.8 µg/mL. 1:1 mixture of phloeodictine C1 and C2. MIC (µg/mL) *Sa* 3, *Ec* > 30, *Pa* > 30, *Cp* > 100, *Bf* > 100 and *Pas* > 100		
phloeodictine C2					
chondropsin A	macrolide lactam	antiproliferative	IC_50_ (nM) KB 1.5, HCT116 1.2, T47D 0.45, HBL100 1.7 and Chang liver 2.4 G2/M cell cycle arrest in HL60 and KB cell lines (→ apoptosis).	*Psammoclemma* sp.	[93]
73-deoxychondropsin A			IC_50_ (nM) KB 0.28, HCT116 0.22, T47D 0.18, HBL100 0.60, Chang liver 0.24 G2/M cell cycle arrest in HL60 and KB cell lines (→ apoptosis).		
echinosulfonic acid D	alkaloid		IC_50_ KB 2 µg/mL.		[94]
psammaplysene C	bromotyrosine alkaloid		IC_50_ THP-1 7 µM.		[95]
psammaplysene D					
reidispongiolide A	sphinxolide-type macrolide	antiproliferative	IC_50_ (µg/mL) KB 0.10, P388 0.16, P388/Dox 0.01, NSCLC-N6 0.07, HT29 0.04	*Reidispongia coerulea*	[83,96]
reidispongiolide B			IC_50_ (µg/mL) KB 0.06, P388 0.06, P388/Dox 0.02, NSCLC-N6 0.05, HT29 0.04		[96]
reidispongiolide C			NCI screening: 60 human cell lines (9 cancer types: leukemia, lung, colon, brain, melanoma, ovarian, renal, prostate and breast). Same degree of cell line selectivity.		[83]
auroral 1	unusual (C(3)-α-OH) truncated isomalabaricane triterpene	antiproliferative	IC_50_ KB 0.2 µg/mL.	*Rhabdastrella globostellata (*formerly *Aurora* sp*.)*	[97]
auroral 2					
auroral 3			IC_50_ KB 8.0 µg/mL.		
auroral 4					
(+)-aeroplysinin-1	bromotyrosine derivative	antibacterial	Active against *Pecten maximus* larvae.	*Suberea creba*	[98]
dibromoverongiaquinol					
demethylxestospongin B	bis-1-oxaquinolizidine alkaloid	antiproliferative somatostatin inhibitor	IC_50_ (µg/mL) KB 2.5, L1210 0.8 No *in vivo* activity against P388 leukemia cells. IP3 active: IC_50_ 12 µM (K_i_ 13.4 µM).	*Xestospongia exigua*	[99]
xestospongin B			IC_50_ (µg/mL) KB 2.5, L1210 2.0. No *in vivo* activity against P388 leukemia cells. IP3 active: IC_50_ 12 µM.		[45,99]
xestospongin D		antiproliferative	IC_50_ (µg/mL) KB 2.0, L1210 0.2 No *in vivo* activity against P388 leukemia cells.		[99]
xestoamine	β-carboline alkaloid		Inactive against KB and L1210 cell lines.		

**Footnote:** **Statistics:** IC_50_ half maximal inhibitory concentration, K_i_ inhibition constant, CD_50_ median cytotoxic dose, ED_50_ median effective dose, SI selectivity index, EC_50_ median effective concentration, IC_90_ inhibitory concentration 90%, MIC minimum inhibitory concentration. **Cell lines:** KB human nasopharyngeal epidermoid carcinoma, L1210 murine lymphocytic leukemia, P388 murine leukemia, P388/Dox murine leukemia doxorubicin-resistant, NSCLC-N6, NSCLC-N6-L16: human non-small-cell bronchopulmonary carcinoma, NSCLC-N6 C15 human non-small-cell bronchopulmonary carcinoma clone 15, NSCLC-N6 C92 human non-small-cell bronchopulmonary carcinoma clone 92, NSCLC-N6 C98 human non-small-cell bronchopulmonary carcinoma clone 98, E39 human renal carcinoma, M96 human melanoma, Jurkat human T leukemia, HBL100, T47D: human breast epithelial, T24 human bladder carcinoma, MCF-7 human breast adenocarcinoma, MCF-7R human breast adenocarcinoma resistant, HCT116, HCT15, HT29: human colon adenocarcinoma, OVCAR8, OV3: human ovary adenocarcinoma, PC3 human prostate adenocarcinoma, Vero monkey kidney, MRC5 fetal human lung, HL60 human promyeocytic leukemia, HL60R human promyeocytic leukemia resistant, K562 human erythromyeloblastoid leukemia, PaCa human pancreas carcinoma, SF268 human glioblastoma, A549 human lung carcinoma, MDA231, MDA435: human breast adenocarcinoma, HepG2 human hepatocarcinoma, EPC epithelioma papulosum cyprini, U266 human multiple myeloma, SKM1 human acute myeloid leukemia, Kasumi human leukemia, THP-1 human monocytic leukemia. **Proteins:** GSK-3β glycogen synthase kinase-3β, SRIF somatotropin release inhibiting factor, VIP vasoactive intestinal peptide, CDC25A protein phosphatase, CDK1 cyclin-dependent kinase 1, CDK2 cyclin-dependent kinase 2, CDK5 cyclin-dependent kinase 5, p25 protein, CK1 casein kinase 1, PLK-1 polo-like kinase 1, PLA_2_ phospholipase A2, NPY neuropeptide Y, TPK tyrosine protein kinase, NK3 tachykinin receptor 3, AMPA α-amino-3-hydroxy-5-methyl-4-isoxazolepropionic acid, CGRP calcitonin gene-related peptide, NT neurotensin. **Viruses:** HIV-1 human immunodeficiency virus type 1; AZT azidothymidine, antiretroviral drug. **Fungi:** *Ca* *Candida albicans*, *Ct Candida tropicalis*, *Pc* *Phythothora citrophthora*, *Pci Phythothora citnnamomi*, *Pr* *Pythium rostatum*, *Pv* *Pythium vexans*, *Bc* *Botrytis cinerea*, *Po* *Pericularia oryzae*, *Fr Fusarium roseum*, *Aa* *Alternaria alternata*, *Rs* *Rhizoctonia solani*, *Ph* *Pseudocercosporella herpotrichoides*, *Sn* *Septoria nodorum*, *Hg Helminthosporium gramineum*, *Un Ustilago nuda*. **Bacteria:** *Sa Staphylococcus aureus*, *Ec Escherichia coli*, *Pf Plasmodium falciparum*, CQ-sensitive *Pf* chloroquine-sensitive *Plasmodium falciparum*, CQ-resistant *Pf* chloroquine-resistant *Plasmodium falciparum*, *Pf* F32, FcB1, PFB: *Plasmodium falciparum* chloroquine-susceptible F32, chloroquine-resistant FcB1, chloroquine-resistant PFB, *Sf Streptococcus faecalis*, *Pa Pseudomonas aeruginosa*, *Cp Clostridium perffingens*, *Bf Bacteroides fragilis*, *Pas* *Peptococcus assaccharolyricus.* **Others:** NO nitric oxide, NCI National Cancer Institute, *N. naja Naja naja,* IP3 inositol-1,4,5-trisphosphate.

**Table 3:** Natural products isolated from New Caledonian ascidians and their bioactivity.

Natural Product	Chemical Class	Biological Activity		Species	Reference
eudistalbin A	β-carboline alkaloid	antiproliferative	ED_50_ KB 3.2 µg/mL.	*Eudistoma album*	[113]
eudistalbin B			Inactive on KB cell line.		
eudistomin E			ED_50_ KB < 5 ng/mL.		
(−)-woodinine	alkaloid	antibacterial	*Sa* 16 mm/100 µg/disk and 18 mm/200 µg/disk. *Ec* 8/100 µg/disk and 11 mm/200 µg/ disk.	*Eudistoma fragum*	[114]
5-bromo-*N,N*-dimethylamino-ethyltryptamine			*Sa* 12 mm/100 µg/disk and 17 mm/200 µg/disk. *Ec* 17 mm/100µg/disk and 22 mm/200 µg/disk.		
bistramide A	tetrahydropyran derivative	antiproliferative	IC_50_ (nM) KB 45, P388 20 and normal human endothelial cells 22 IC_50_ NSCLCN6-L16 0.49 µM at 67 h. IC_50_ (µg/mL) KB 0.53, P388 0.20, P388/Dox 0.05, B16 0.10, HT29 0.32, NSCLC-N6 0.03	*Lissoclinum bistratum*	[115,116,117,118,119,120]
		antibacterial	Inactive agaisnt *Ec*, *Kp*, *Mm*, *Pm*, *Pv*, *Pa*, *Sm*, *Sa* and *Streptococcus* group D (500 µg/mL).		
		mechanism of action	G1 cell cycle arrest in NSCLCN6-L16 cells; polyploidy-inaptitude for cytokinesis.		
		Na^+^ channels inhibitor	At rest and in the inactivated state, occupied a site which was not located on the inactivation gate.		
		Ca^2+^ sensitivity immunomodulator	Binding to contractile proteins for which it competes with Ca^2+^. Inhibition of T cell proliferation and activation of B cell proliferation.		
bistramide B		antiproliferative mechanism of action	IC_50_ (µg/mL) KB 2.10, P388 0.20, P388/Dox 1.16, B16 1.20, HT29 0.71, NSCLC-N6 0.32. Significant decreases of S phase in NSCLC-N6 cells.		[117]
bistramide C			IC_50_ (µg/mL) KB 0.65, P388 0.02, P388/Dox 0.05, B16 0.06, HT29 0.50, NSCLC-N6 0.05. Significant decreases of S phase in NSCLC-N6 cells.		
bistramide D			IC_50_ (µg/mL) KB 10.00, P388 0.36, P388/Dox 5.82, B16 0.10, HT29 2.76, NSCLC-N6 3.43; *In vivo* (IV and IP) antitumor activity in nude mice engrafted SC with NSCLC-N6, T/C 53% at day 30. Significant decreases of S phase; partial G1 cell cycle arrest in NSCLC-N6 cells.		
bistramide K	tetrahydropyran derivative	antiproliferative mechanism of action	IC_50_ (µg/mL) KB > 10.00, P388 0.57, P388/Dox > 10.00, B16 1.90, HT29 5.60, NSCLC-N6 3.23; *In vivo* (IV and IP) antitumor activity in nude mice engrafted SC with NSCLC-N6, T/C 49% at day 30. G1 cell cycle arrest in NSCLC-N6 cells.	*Lissoclinum bistratum*	[117]
dichlorolissoclimide	nitrogenous labdane diterpene	antiproliferative mechanism of action	IC_50_ (ng/mL) KB 14, P388 1, P388/Dox 300 and NSCLC-N6 9; G1 cell cycle arrest in NSCLC-N6 cells (irreversible, total, dose-and time-dependent).	*Lissoclinum voeltzkowi*	[121,122]
chlorolissoclimide		antiproliferative mechanism of action	IC_50_ (ng/mL) KB 52, P388 1.7, P388/Dox 200 and NSCLC-N6 10; G1 cell cycle arrest in NSCLC-N6 cells.		[121,122,123]
arborescidine D	indole alkaloid	antiproliferative	IC_50_ KB 3 µg/mL.	*Pseudodistoma arborescens*	[124]

**Footnote:** **Statistics:** ED_50_ median effective dose, IC_50_ half maximal inhibitory concentration, T/C tumor growth inhibition ratio. **Cell lines:** KB human nasopharyngeal epidermoid carcinoma, P388 murine leukemia, P388/Dox murine leukemia doxorubicin-resistant, NSCLCN6-L16, NSCLC-N6: human non-small-cell bronchopulmonary carcinoma, B16 murine melanoma, HT29 human colon adenocarcinoma. **Bacteria:** *Sa* *Staphylococcus aureus*, *Ec* *Escherichia coli*, *Kp Klehsiella pneumoniae*, *Mm Morganella morganii*, *Pm Proteus mirabilis*, *Pv Proteus vulgaris*, *Pa Pseudomonas aeruginosa*, *Sm Serratia mareescens*. **Ions:** Na^+^ sodium, Ca^2+^ calcium. **Others:** IV intravenous, IP intraperitoneal, SC subcutaneous.

**Table 4:** Natural products isolated from New Caledonian Cnidaria and their bioactivity.

Natural Product	Chemical Class	Biological Activity		Species	Reference
7-*epi*-11,19-desoxyhavannahine	xenicane diterpene	anti-fouling	*Ceramium codii* RGR after 2 days: 4% of control at 50 ppm, 21% at 25 ppm, 42% at 12.5 ppm.	*Xenia garciae*	[33]
*iela melst*	protein	elastase inhibitor	Inhibitor of amidolysis of Suc(Ala)3p-NA by porcine pancreatic elastase (K_i_ 1.5 nM).	*Melithea* cf. *stormii*	[37]
villogorgin A	caffeine-xanthine type alkaloid	anti-inflammatory	Acetylcholine antagonist. Anti-aggregatory (thrombin, A23187).	*Villogorgia rubra*	[127]
lituarine A	polyethermacrolide	antiproliferative	IC_50_ KB 3.7–5 ng/mL.	*Lituaria australasiae*	[128]
lituarine B			IC_50_ KB 1–2 ng/mL.		
lituarine C			IC_50_ KB 5–6 ng/mL.		
pteroidin	briarane diterpene	ichtyotoxic	LD_100_ 50 µg/mL, *t* = 90 min (fish of the genus *Mugil*).	*Pteroides laboutei*	[129]
*O*-deacetyl-12-*O*-benzoyl-12-pteroidin			LD_100_ 50 µg/mL, *t* = 150 min (fish of the genus *Mugil*).		

**Statistics:** RGR Relative Growth Rate, K_i_ inhibition constant, IC_50_ half maximal inhibitory concentration, LD_100_ lethal dose 100%. **Cell line:** KB human nasopharyngeal epidermoid carcinoma. **Other:** A23187 calcium ionophore.

**Table 5:** Natural products isolated from New Caledonian Echinodermata and their bioactivity.

Natural Product	Chemical Class	Biological activity		Species	Reference
gymnochrome B	phenanthroperylene-quinone pigment	antiviral	DENV RF_50%_ 1 µg/mL.	*Gymnochrinus richeri*	[141,142]
gymnochrome D			DENV RF_50%_ < 1 µg/mL.		
isogymnochrome D					
ptilomycalin A	guanidine alkaloid	antiviral	IC_50_ HIV-1 0.11 µg/mL. DENV inactive.	*Celerina heffernani*	[142,143]
celeromycalin			IC_50_ HIV-1 0.32 µg/mL. DENV inactive.		
fromiamycalin			IC_50_ HIV-1 0.11 µg/mL.	*Fromia monolis*	
crambescidin 800			IC_50_ HIV-1 0.11 µg/mL. DENV inactive.		
(25S)-5α-cholestane-3β,4β,6β, 7α,8,15α,16β,26-octol	sterol	antifungal	Active at 5 µg against *Clodosporium cucumerinum.*	*Rosaster* sp.	[144]

**Footnote: Viruses:** DENV dengue virus, HIV-1 human immunodeficiency virus type 1. **Statistics:** RF_50%_ reduction factor 50%, IC_50_ half maximal inhibitory concentration.

**Table 6:** Subsequent and ongoing development of selected natural products isolated from New Caledonian marine organisms.

Natural Product and/or Analogs	Chemical Synthesis and Biological Activity	Reference
(−)-agelastatin A–F	-Enantioselective total synthesis involving late-stage C-ring formation. -(−)-agelastatin A: antiproliferative activity highly potent on blood cancer cell lines (CEM EC_50_ 20 nM; Jurkat EC_50_ 74 nM; Daudi EC_50_ 20 nM; HL-60 EC_50_ 138 nM; CA46 EC_50_ 187 nM) *vs.* normal red blood cells (EC_50_ > 333 μM); dose-dependent induction of apoptosis (G2/M phase arrest), no effect on tubulin dynamics.	[181]
agelastatin A/13-debromo-13-trifluoromethyl agelastatin A	-*In vitro* and *in vivo* antiproliferative activity: CLL patient (CLL1 and CLL2) and JVM-2 cell line (EC_50_ 0.064 µM and 0.16 µM respectively). -Chemical modifications outside the pyrrole ring result in significant loss in activity. -Importance of an electronegative functional group at position C-13 for CLL activity.	[182]
suvanine	-Anti-inflammatory activity, Hsp 60 inhibitor.	[183]
	-Antagonist of farnesoid-X-receptor (FXR). -Identification of conformational changes responsible for agonist/antagonist form on FXR using suvanine as template.	[184]
	-Inhibitor of hepatitis C virus NS3 helicase (IC_50_ of 3 µM). -Inhibition of ATPase, RNA binding, and serine protease activities of NS3 helicase with IC_50_ 7, 3, and 34 µM, respectively. -Interaction with an allosteric site in NS3 rather than binding to the catalytic core.	[185]
isohymenialdisine and hymenialdisine	-Inhibition of CLK kinases (hymenialdisine only).	[186]
	-Stimulation of translation: isohymenialdisine and hymenialdisine act on PKR (RNA-dependent protein kinase) by inhibiting its autophosphorylation and pertub the PKR-eIF2α phosphorylation axis; models indicate that it fits in the PKR ATP binding site.	[187]
	-Short (6 steps), concise, and high yielding (44%) total synthesis of hymenialdisine will enable the synthesis of novel libraries for subsequent SAR studies.	[188]
arsenicin A	-Synthesis of sulfur-derivatives. -Antiproliferative activity of monosulfide (±)-arsenicin A (more potent on APL cells than (±)-arsenicin A and arsenic (III) oxide); induction of apoptosis.	[189]
heteronemin	-Anti-intravasative properties.	[190]
	-Targets TDP-43, binds to specific sequences on DNA and RNA.	[191]
	-Antiproliferative (A498 EC_50_ 1.57 μM). -Induction of both apoptosis and autophagy (heteronemin inhibits the phosphorylation of ERK and AKT signaling pathways and increases the phosphorylation of p38 and JNK).	[192]
bengamides and analogs	-Synthesis of novel caprolactam-ring-opened bengamide analogs with antitumor activity on MDA-MB-435 (compounds 3a (EC_50_ 4 nM) and 2i (EC_50_ 9 nM) more potent activity than LAF389, analog of bengamide (EC_50_ 40 nM) and the original caprolactam analog 10’ (EC_50_ 17 nM). -Improved water solubility.	[193]
	Methionine aminopeptidases inhibitors (HsMetAP1 and HsMetAP2).	[194]
	-Synthesis of a series of inhibitors of methionine aminopeptidases of *Mycobacterium tuberculosis.* -New X-ray structures of MtMetAP1c in complex with inhibitors in Mn(II) and Ni(II) forms; all amide moieties bind to the unique shallow cavity and interact with flat surface created by His-212 of MtMetAP1c in the Mn(II) form. Influence of active site metal on binding mode (amide takes on a different conformation in the Ni(II) form).	[195]
	-Analogs of bengamide E with antiproliferative properties (modified at the terminal olefinic position). -More potent activity: compound 56 (a cyclopentyl group replaced the isopropyl group at the terminal olefinic position).	[196]
	-Synthesis of stereoisomers of bengamide E (2,3-bis-*epi*- and the 2-epianalogues), a collection of C2-modified analogues, and various epoxy bengamides. -Stereochemistry at C2 and C3 positions and methoxyl group at C2: essential for retaining the cytotoxic potency.	[197]
naamidine A	-Antitumor agent; induction of apoptosis (caspase-dependent).	[198,199]
microsclerodermin A	-Inhibition of NFκB transcriptional activity, reduced levels of phosphorylated (active) NFκB in the AsPC-1 cell line. -Antiproliferative activities: AsPC-1, BxPC-3, MIA PaCa-2 and PANC-1 pancreatic cancer cell lines. -Induction of apoptosis in the AsPC-1 mediated by GSK-3β pathway.	[200]
petrosaspongiolide M and analogs	-Synthetic derivative of benzo[*b*]thiophen-2-yl-3-bromo-5-hydroxy-5*H*-furan-2-one. -*In vitro* and *in vivo* potent anti-inflammatory activity (inhibition of NF-κB signaling pathway and STAT3 phosphorylation).	[201]
	-Proteasome inhibitor. -Inhibitory activity by binding the active sites in the inner core of the immunoproteasome and/or covalently linking a Lys residue at the proteasome core/11S activator particle interface. -Modulation of intracellular proteolysis through a dual inhibition of the immunoproteasome and autophagy.	[202]
aeroplysinin-1	-Induction of apoptosis in endothelial cells (caspase dependent).	[203]
	-Antiproliferative effect on acute myeloid leukemia (AML) cells (dose-dependent EC_50_ 10–20 μM); induction of apoptosis. -Agent-specific pleiotropic effects.	[204]
fistularin-3	-Antiproliferative activity dose- and time-dependent (EC_50_ 7.39 and 8.10 μM for Jurkat E6.1 and U937 resp.); Pro-apoptotic.	[205]
Analogs of bistramide A	-Design and synthesis of analog of bistramide A targeting cytoskeletal organization of cancer cells *in vivo* (combination of reversible G-actin binding and effective F-actin severing). -Potent and reversible binding of monomeric actin (K_d_ 9.0 nM), *in vitro* depolymerization of actin; *in vitro* and *in vivo* inhibition of A549 cells.	[206]
	-Computational analyses of non-covalent actin-inhibitor interactions (AutoDock and DrugScore scoring function). -Design of a novel, highly modular class of hybrid analogs with a rationale to address both the bistramide and rhizopodin binding sites. -Antiproliferative activity is conserved in analogs resembling the original side chain of rhizopodin.	[207]
luzonicoside A	-Potent immunomodulatory agent: more effective in stimulating lysosomal activity, intracellular ROS level elevation, and NO synthesis up-regulation in RAW 264.7 murine macrophages cells (0.01–0.1 μM).	[208]

**Cell lines:** CEM human acute lymphoblastic leukemia, Jurkat human T leukemia, Daudi human B lymphoma (Burkitt’s lymphoma), HL-60 human acute promyelocytic leukemia, CA46 human lymphoma (Burkitt’s lymphoma), CLL chronic lymphocytic leukemia, JVM-2 human lymphoma (Mantle Cell Lymphoma), APL acute promyelocytic leukemia, A498 human kidney carcinoma, MDA-MB-435 human breast adenocarcinoma, AML acute myeloid leukemia, AsPC-1, BxPC-3, MIA Paca-2, PANC-1 pancreatic cancer cell lines. **Proteins:** Hsp-60 Heat shock protein 60, FXR farnesoid X receptor, NS3 nonstructural protein 3, CLK protein kinases, PKR protein kinase R, TDP-43 TAR (trans-activator regulatory) DNA-binding Protein 43, ERK extracellular signal-regulated kinase, AKT protein kinase B, JNK *c*-Jun N-terminal kinase, GSK-3β glycogen synthase kinase-3β, STAT3 signal transducer and activator of transcription. **Virus:** HCV hepatitis C virus. **Others:** SAR structure-activity relationship, NO nitric oxide.
